# Macrophage Lipid Homeostasis Drives IVDD via a Senescence‐Dependent Impairment of Efferocytosis

**DOI:** 10.1111/cpr.70271

**Published:** 2026-08-19

**Authors:** Jinyu Wang, Shiyong Ling, Qi Wang, Xuege Qiao, Jianpeng Xing, Siyang Ji, Linhui Han, Chen Yan, Zhishen Niu, Lei Yuan, Baolian Zhao, Kaiqiang Sun, Jiangang Shi

**Affiliations:** ^1^ Department of Orthopedic Surgery Changzheng Hospital, Naval Medical University Shanghai P. R. China; ^2^ Department of Orthopaedics Shanghai Jing'an District Zhabei Central Hospital Shanghai China; ^3^ School of Basic Medical Sciences, Naval Medical University Shanghai China; ^4^ Department of Health Management, Faculty of Military Health Service Naval Medical University Shanghai China; ^5^ Department of Radiology Changzheng Hospital, Naval Medical University Shanghai China; ^6^ Department of Orthopedics Naval Medical Center of PLA Shanghai P. R. China

**Keywords:** cellular senescence, efferocytosis, intervertebral disc degeneration, lipophagy, macrophage lipid homeostasis, rosuvastatin

## Abstract

Intervertebral disc degeneration (IVDD) is a major contributor to low back pain, but the immune‐metabolic events that sustain disc inflammation remain poorly defined. Here, we investigated whether disturbed macrophage lipid handling promotes IVDD by coupling cellular senescence to defective efferocytosis. Clinical magnetic resonance imaging and biochemical profiling showed that advanced IVDD was accompanied by increased disc fat fraction and systemic lipid abnormalities. Mendelian randomisation, bulk transcriptomics, machine‐learning modelling and single‐cell RNA sequencing further linked lipid metabolic disturbance to immune remodelling in degenerative discs, and identified TIAM2, SLC44A4, PPT1 and PTGDS as lipid metabolism‐related hub genes associated with IVDD. At the single‐cell level, disc macrophages with low glycerophospholipid metabolism scores showed higher senescence activity, a more inflammatory polarisation state and reduced efferocytosis‐related signatures. Functional studies in bone marrow‐derived macrophages indicated that lipid overload promoted lipid droplet accumulation, lipid peroxidation, impaired lipophagy and cholesterol efflux, activation of the p53/p21 senescence pathway, and reduced apoptotic nucleus pulposus cell clearance. Restoring lipid homeostasis with rosuvastatin enhanced lipophagy, recovered ABCA1/ABCG1‐mediated cholesterol export, attenuated macrophage senescence and inflammatory activation and improved efferocytosis. In a rat needle puncture model, local rosuvastatin delivery alleviated disc structural damage, preserved proteoglycan content, balanced extracellular matrix metabolism and reduced macrophage senescence and inflammatory markers. In conclusion, this study supports macrophage lipid homeostasis as an important regulator of IVDD‐associated immune dysfunction through senescence‐associated impairment of efferocytosis.

## Graphical abstract

Intervertebral disc degeneration (IVDD) has been a leading cause of low back pain and lacks disease‐modifying therapies. Although dysregulated disc lipid metabolism is implicated, its impact on the immune microenvironment, particularly macrophages, remains unknown. This study aimed to elucidate the role of macrophage lipid homeostasis in IVDD by testing the hypothesis that metabolic dysregulation drives macrophages into dysfunctional senescence. We integrated multidimensional evidence: clinical and multi‐omics analyses established causality between lipid disorders and IVDD; machine learning and single‐cell RNA sequencing identified hub genes (TIAM2, SLC44A4, PPT1, PTGDS) and macrophage subsets. In vitro experiments validated the lipotoxicity–senescence–inflammation (LSI) axis. Animal models evaluated rosuvastatin's therapeutic potential. Results demonstrated that IVDD progression is driven by a self‐amplifying LSI axis. Macrophages with low lipid metabolism scores exhibited high senescence, proinflammatory phenotype and impaired efferocytosis. Rosuvastatin restored lipophagy and cholesterol efflux, alleviating senescence, improving efferocytosis and reducing inflammation, which was confirmed in a rat IVDD model where local treatment ameliorated degeneration and immune dysregulation. Our findings established macrophage lipid homeostasis as a linchpin in IVDD pathology and identify statin‐mediated targeting of the LSI axis as a translatable immunometabolic strategy.

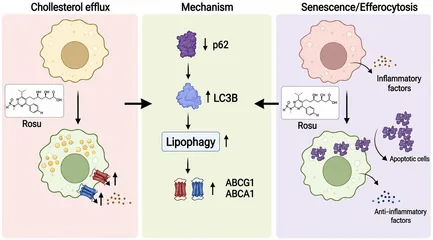

## 1 Introduction

Intervertebral disc degeneration (IVDD) is a leading global cause of debilitating low back pain (LBP) and imposes a substantial socioeconomic burden worldwide [1]. Epidemiological studies indicate that most adults experience LBP at least once during their lifetime, and severe cases can result in mobility loss and long‐term disability [2, 3]. Current clinical management primarily provides symptomatic relief but does not halt or reverse the degenerative process [4]. Therefore, defining the mechanisms that drive IVDD progression is essential for developing disease‐modifying therapies.

The healthy intervertebral disc, comprising the annulus fibrosus (AF), nucleus pulposus (NP), and endplates (EP), is located between adjacent vertebrae [5]. Its gelatinous NP core disperses mechanical loads and absorbs axial stress, thereby enabling spinal articulation and load buffering [6]. Although the pathogenesis of IVDD remains incompletely understood, NP cell loss and extracellular matrix (ECM) imbalance are widely recognised as central events in disease progression [7]. However, therapeutic strategies focused solely on NP cell regeneration have achieved limited clinical efficacy [8]. Increasing evidence now highlights the degenerative disc as an immune‐active microenvironment in which inflammatory remodelling critically shapes tissue degeneration [4, 5, 9, 10, 11]. Macrophages, as highly plastic innate immune cells, regulate inflammation, tissue clearance, and repair through dynamic changes in polarisation and efferocytosis, and these phenotypes are tightly controlled by extracellular cues and cellular metabolism [4, 12, 13, 14].

Macrophage function is profoundly influenced by cellular metabolism, and lipid metabolic pathways regulate inflammatory cytokine production and antiviral responses in macrophages [12, 15, 16]. Conversely, macrophage lipid dysregulation contributes to multiple inflammatory and degenerative diseases, including atherosclerosis and immune‐mediated disorders [17]. Aberrant lipid accumulation has also been observed in IVDD [18]. After lipid uptake, macrophages degrade lipids into free cholesterol and fatty acids within lysosomes and transport these products to the endoplasmic reticulum for esterification and storage as lipid droplets [17]. Pro‐inflammatory M1 macrophages typically exhibit a glycolysis‐dominant metabolic program accompanied by suppression of fatty acid oxidation (FAO) and oxidative phosphorylation (OXPHOS) [19, 20]. In contrast, M2 macrophages rely primarily on FAO for energy supply and maintain intact lipid metabolic homeostasis [15, 21], whereas anti‐inflammatory M2 macrophages rely more heavily on FAO and maintain lipid metabolic homeostasis [22]. Importantly, intracellular lipid overload can trigger cellular senescence through pathways such as LXR/CD38‐mediated NAD^+^ depletion or PPARα inhibition [22]. Senescent macrophages not only lose homeostatic functions but also acquire a senescence‐associated secretory phenotype (SASP) that sustains inflammation and propagates senescence signals [23]. These observations raise several key questions in IVDD: whether macrophage lipid homeostasis becomes dynamically disrupted, which microenvironmental cues initiate this shift, and how metabolic reprogramming impairs efferocytosis and resolution functions, converting macrophages from tissue guardians into drivers of degeneration.

We therefore hypothesised that lipid metabolic dysregulation within the IVDD microenvironment acts as a pathogenic trigger that drives macrophages into a dysfunctional senescent state. This state may constitute the central node of a self‐amplifying Lipotoxicity‐Senescence‐Inflammation (LSI) axis. To test this hypothesis, we designed a multidimensional, causality‐oriented strategy. We integrated clinical imaging and Mendelian randomisation to examine lipid‐IVDD associations, applied machine learning and single‐cell transcriptomics to identify key regulators and macrophage states, used in vitro functional assays to dissect the LSI axis, and evaluated statin‐based modulation of this axis in vivo [24]. This study aims to establish a mechanistic framework linking lipid imbalance, macrophage senescence, defective efferocytosis and immune failure in IVDD.

## 2 Methods and Materials

### 2.1 Human Ethics

Human disc tissues, imaging data, and clinical laboratory data were obtained from discarded clinical specimens or routine clinical examinations, with no additional intervention performed for research purposes. All human specimens and data were fully anonymised before analysis. Because this study involved no human intervention and posed minimal ethical risk, ethical approval was waived by the Institutional Review Board, and written informed consent was not required. All animal experiments were approved by the Institutional Animal Care and Use Committee of Naval Medical University and conducted in accordance with institutional guidelines.

### 2.2 Evaluation of Lumbar Disc Status and Fat Fraction Using T2WI Combined With IDEAL‐IQ Fat Fraction Imaging

T2‐weighted imaging (T2WI) reflects differences in tissue T2 relaxation time, with signal intensity primarily related to free water content, collagen distribution, and microstructural organisation. IDEAL‐IQ (Iterative Decomposition of Water and Fat with Echo Asymmetry and Least‐Squares Estimation‐Quantitative Imaging) is a chemical shift‐based water‐fat separation technique for quantitative fat assessment. Fat fraction (FF) was defined as the ratio of fat proton signal intensity to the total signal intensity of water and fat protons in a single voxel and was calculated as FF = (M_fat_/(M_fat_ + M_water_)) × 100% [25].

### 2.3 Single‐Cell Sequencing Analysis

Single‐cell RNA sequencing (scRNA‐seq) data were processed and analysed using the Seurat package. Normalisation and scaling were performed with the Seurat functions NormalizeData and ScaleData to reduce potential confounding effects, including cell‐cycle phase and mitochondrial gene proportion. Uniform Manifold Approximation and Projection (UMAP) was used for dimensionality reduction and visualisation. Cell identities were assigned by integrating cluster‐specific markers with previously reported cell‐type marker databases, enabling downstream analysis of cellular heterogeneity and biological function [26].

### 2.4 Glycerophospholipid Metabolism Score, Senescence Score and Polarisation Score

To quantify macrophage metabolic activity and senescence status at the single‐cell level, we calculated glycerophospholipid metabolism (GPL), senescence, and polarisation scores. For each cell, single‐sample gene set enrichment analysis (ssGSEA), implemented in the R package GSVA (v1.44.0), was used to integrate the expression levels of pathway‐specific genes and generate continuous enrichment scores reflecting the relative activity of glycerophospholipid metabolism, cellular senescence, and macrophage polarisation. Score distributions were visualised using violin plots generated with the R package vioplot (v0.3.7).

### 2.5 Mendelian Randomisation (MR) Design

#### 2.5.1 Data Sources

Mendelian randomisation (MR) enables reliable inference of the causal association between exposure factors and outcome variables (e.g., diseases) in observational data by utilising genetic variations as ‘instrumental variables’. In this two‐sample genome‐wide association study (GWAS), genetic variant data related to inflammatory proteins and lipidomics were acquired through large‐scale GWAS analyses. Specifically, the dataset included 91 inflammatory proteins and 179 lipids, whose corresponding GWAS data are accessible from the EBI GWAS Catalogue (http://ftp.ebi.ac.uk/) [27, 28]. The statistical data regarding intervertebral disc degeneration (IVDD) was retrieved from FinnGen Consortium R10, which was released in 2023 (www.finngen.fi/en). This dataset comprised 41,669 cases, 294,770 controls, and a total of 21,311,942 single nucleotide polymorphisms (SNPs).

#### 2.5.2 Instrumental Variables (IVs) Selection

(a) The filtering threshold for SNPs associated with the 91 inflammatory proteins and 179 lipids was each set at *p* < 5 × 10^−6^. (b) A minimum physical distance of 10,000 base pairs was required between any two SNPs, and the linkage disequilibrium *r* ^2^ threshold among genes was set to less than 0.001. Palindromic SNPs with inconsistent effect allele frequencies between the exposure and outcome datasets were eliminated. (c) SNPs were included only if their *F*‐statistic exceeded 10. (d) The PhenoScanner tool was utilised to exclude SNPs that might introduce confounding effects.

#### 2.5.3 Sensitivity Analysis

Heterogeneity was assessed via the *p* value derived from Cochran's *Q* test, with a *p* value < 0.05 indicating the presence of significant heterogeneity. The MR‐Egger intercept test was applied to detect potential horizontal pleiotropy. The leave‐one‐out analysis was conducted to identify outlier SNPs and verify the stability of the results by sequentially removing each SNP and re‐evaluating the outcomes [29].

#### 2.5.4 MR Analysis Procedures

A two‐step MR strategy was employed for mediated MR analysis. All MR analyses were carried out using the ‘TwoSampleMR’ R package in R version 4.3.1, with the inverse variance weighting (IVW) method serving as the primary approach to estimate the causal relationship between exposures and outcomes. Additionally, MR‐Egger, weighted median, weighted mode, and simple mode methods were utilised to validate the robustness of the obtained results.

### 2.6 Consensus Cluster Plus‐Based Clustering Analysis

Transcriptomic data from intervertebral disc tissues of IVDD patients (accession number: HRA009301) were downloaded from the GSA repository (https://ngdc.cncb.ac.cn/gsa‐human). A total of 122 IVDD samples with complete data were included. Lipid metabolism‐related genes (LMGs) were obtained by integrating the GeneCards database (https://www.genecards.org/) with prior lipid metabolism studies. Genes with zero expression in at least 70% of samples were excluded. Consensus clustering was then performed using the R package ConsensusClusterPlus (v1.60.0) to evaluate subgroup stability across different cluster numbers [30].

### 2.7 Identification of IVDD‐Associated Lipid Metabolism Hub Genes via Machine Learning Models

Multiple IVDD‐related transcriptomic datasets (GSE153761, GSE56081 and GSE70362) were integrated to extract gene expression matrices from normal and degenerative intervertebral disc tissues. After integrating the LMG set with previously reported lipid metabolism genes, differentially expressed genes (DEGs) between IVDD and control tissues were identified using a threshold of *p* < 0.05. The intersection of LMGs and DEGs was defined as the candidate feature set. Genes with at least 30% missing values were removed. The feature matrix was standardised by min‐max normalisation in R (v4.2.1), and remaining missing values were imputed using the random forest algorithm. For LASSO regression, the regularisation parameter *λ* was selected by cross‐validation to minimise prediction error and reduce model complexity. The initial LASSO model was constructed using the integrated discovery dataset and was used primarily for candidate feature selection. No independent external validation cohort was available for this initial LASSO screening step; therefore, the AUC derived from this model was interpreted as internal apparent performance rather than definitive diagnostic accuracy. To improve robustness, the LASSO‐selected genes were further evaluated using CatBoost, LightGBM, and XGBoost models, and SHAP analysis was applied to identify genes consistently contributing to model prediction across algorithms.

### 2.8 Machine Learning Algorithms and SHAP Analysis

Three ensemble learning algorithms, CatBoost, LightGBM, and XGBoost, were used to establish IVDD risk prediction models. The CatBoost model (R package CatBoost v1.2.2) was configured with 1000 iterations, a learning rate of 0.05, tree depth of 6, l2_leaf_reg of 3, and classification as the task type. For the LightGBM model (R package LightGBM v1.1.5), the base learner was DecisionTreeRegressor, with 800 estimators, a learning rate of 0.08, a maximum tree depth of 5, and a Bernoulli distribution for binary classification. The XGBoost model (R package xgboost v1.7.5.1) was configured with 1200 rounds, eta = 0.03, maximum tree depth = 4, lambda = 1, alpha = 0.5, subsample = 0.8, colsample_bytree = 0.7, and the binary:logistic objective function [31].

### 2.9 Gene Set Enrichment Analysis (GSEA)

Gene Set Enrichment Analysis (GSEA) was conducted as follows: the FindMarkers function was utilised to identify DEGs between the control and IVDD groups in the three metabolic clusters prior to performing GSEA. The significance threshold was set at an adjusted *p* value < 0.05 [32].

### 2.10 Survival Analysis Curve

Data were extracted from the UK Biobank (UKB), a large prospective cohort. Eligible participants were those with recorded use of lipid‐lowering agents, including statins, fibrates, cholesterol absorption inhibitors, or combined regimens. Individuals with baseline IVDD, incomplete medication data, or loss to follow‐up were excluded. The exposure variable was lipid‐lowering drug class, and the primary outcome was incident IVDD, defined by ICD‐10 code M51.x. Survival curves were generated using the Kaplan–Meier method, and differences among groups were assessed using log‐rank tests. Age, sex, BMI, and comorbidities were adjusted as covariates. Analyses were performed using R (v4.3.1) with the survival and survminer packages, and *p* < 0.05 was considered statistically significant. This research was conducted using the UK Biobank Resource under Application Number 99709.

### 2.11 Western Blotting (WB) Assay

Proteins were extracted from cultured cells using radioimmunoprecipitation assay (RIPA) buffer and quantified with a bicinchoninic acid (BCA) assay kit. After heat denaturation, proteins were separated by SDS‐PAGE and transferred onto polyvinylidene fluoride (PVDF) membranes. Membranes were blocked with 5% skimmed milk and incubated with primary antibodies overnight at 4°C (Immunoway, Product Nos. YM8147, YN0996, YT5060 and YN2847), followed by incubation with secondary antibodies for 1 h at room temperature (Immunoway, Product No. RS0006). Band intensities were quantified using ImageJ (v1.8.0), normalised to GAPDH, and expressed as relative grey values.

### 2.12 Primary RT‐PCR and Quantitative Analysis

Total RNA was extracted from bone marrow‐derived macrophages (BMDMs) using a rapid RNA isolation kit (Magen, Guangzhou, China) according to the manufacturer's instructions. Subsequently, 1–2 μg of total RNA was reverse‐transcribed into complementary DNA (cDNA) using a reverse transcription kit (TaKaRa Biotechnology, Otsu, Japan). Quantitative real‐time polymerase chain reaction (qRT‐PCR) was performed using SYBR Premix Ex Taq (TaKaRa Biotechnology) on a real‐time PCR system (Applied Biosystems, Foster City, CA, USA). Thermal cycling consisted of 40 cycles of denaturation at 95°C for 10 s followed by annealing/extension at 60°C for 30 s. Each reaction was performed in triplicate, and relative gene expression was normalised to glyceraldehyde‐3‐phosphate dehydrogenase (GAPDH).

### 2.13 Isolation and Culture of Bone Marrow‐Derived Macrophage (BMDM) Cells

In the present study, bone marrow‐derived macrophages (BMDMs) were isolated from the femurs and tibias of 4–6‐week‐old mice. Mice were euthanized with isoflurane, after which their femurs and tibias were harvested on a sterile laminar flow hood with the adherent muscle tissues carefully removed. The isolated bones were immersed in 75% ethanol for 10 min for disinfection, followed by a 5‐min immersion in phosphate‐buffered saline (PBS) supplemented with 1% penicillin–streptomycin (P/S) and 30 ng/mL macrophage colony‐stimulating factor (M‐CSF). Subsequently, both ends of the bilateral femurs and tibias were cut open with sterile scissors. The bone marrow cavity was thoroughly flushed with complete α‐minimum essential medium (α‐MEM) (containing 10% fetal bovine serum (FBS), 1% P/S and 30 ng/mL M‐CSF) using a sterile 5 mL syringe, and the harvested bone marrow suspension was gently dissociated into a single‐cell suspension. The single‐cell suspension was then seeded into 25 cm^2^ T‐flasks and cultured for 3 days in a humidified incubator at 37°C with 95% air and 5% CO_2_. Under the stimulation of M‐CSF, only BMDMs were capable of adhesion and survival. The obtained BMDMs were either subcultured for subsequent passages or directly used for follow‐up experimental assays [33].

### 2.14 Oil Red O Staining

Intracellular lipid accumulation was evaluated using Oil Red O staining. Briefly, bone marrow‐derived macrophages were seeded onto sterile coverslips or culture plates and treated as indicated. After treatment, cells were washed twice with PBS and fixed with 4% paraformaldehyde for 15–20 min at room temperature. The cells were then washed with PBS and stained with freshly prepared Oil Red O working solution for 15–30 min. After staining, excess dye was removed, and the cells were washed with distilled water. Lipid droplets were observed under a light microscope and appeared as red‐stained intracellular deposits. The Oil Red O‐positive area or staining intensity was quantified using ImageJ.

### 2.15 Lipid Peroxidation Staining

Lipid peroxidation was assessed using a BODIPY 581/591 C11‐based lipid peroxidation assay kit according to the manufacturer's instructions. Briefly, bone marrow‐derived macrophages were seeded onto coverslips or culture plates and treated as indicated. Cells were washed twice with PBS and incubated with freshly prepared BODIPY 581/591 C11 working solution at 37°C for 30 min in the dark. After washing with PBS, cells were immediately observed under a fluorescence microscope. Oxidised and non‐oxidised lipids were detected in the green and red fluorescence channels, respectively. Lipid peroxidation was quantified as the ratio of green to red fluorescence intensity using ImageJ.

### 2.16 Senescence Staining

Cellular senescence was assessed using a senescence‐associated β‐galactosidase staining kit. Briefly, bone marrow‐derived macrophages were seeded onto sterile coverslips or culture plates and treated as indicated. Cells were washed twice with PBS, fixed for 10–15 min at room temperature, and then incubated with freshly prepared β‐galactosidase staining solution at 37°C overnight in a CO_2_‐free incubator. After staining, cells were washed with PBS and observed under a light microscope. Cells with blue cytoplasmic staining were considered senescent cells. The percentage of SA‐β‐gal‐positive cells was calculated as the number of blue‐stained cells divided by the total number of cells.

### 2.17 Efferocytosis Assay

Briefly, BMDM were seeded in 6‐well plates at a density of 1 × 10^6^ cells per well and maintained overnight in DMEM supplemented with 10% FBS. Cells were subsequently treated for 24 or 48 h with lipopolysaccharide (LPS, 10 ng/mL) and apoptotic NPCs. Apoptotic NPCs were pre‐induced by stimulation with TNF‐α (500 ng/mL) for 24 h. CFSE‐labelled apoptotic NPCs were co‐cultured with macrophages at a 10:1 ratio for 120 min. Following incubation, cells were thoroughly washed three times with PBS to remove non‐phagocytosed NPCs. Efferocytosis was assessed using immunofluorescence microscopy. The efferocytosis index was quantified as the percentage of macrophages containing apoptotic bodies relative to the total macrophage count, multiplied by 100%.

### 2.18 Establishment of the IVDD Animal Model

All procedures for establishing the IVDD rat model were conducted in compliance with institutional animal care and use guidelines. Eight‐week‐old SD rats were employed to generate a needle puncture‐induced IVDD model. Briefly, after skin preparation, rats were anaesthetised with isoflurane. A 22 G needle was vertically inserted into the caudal 5/6 intervertebral disc, rotated 180 degrees, and held in place for 10 s. For the in vivo rat IVDD model, rosuvastatin was administered by local intradiscal injection at a concentration of 10 ng/mL in a volume of 5 μL. This low‐dose local delivery strategy was chosen because the intervertebral disc is a confined anatomical compartment, and direct intradiscal injection allows the drug to act locally at the degenerative lesion while minimising potential systemic exposure. The injection volume of 5 μL was selected based on our previous experience with local intradiscal administration, as this volume is sufficient to deliver the therapeutic dose while avoiding obvious leakage from the disc space [34]. Therefore, the final dose was determined by considering both previous in vivo rosuvastatin studies and the technical feasibility of local intradiscal injection. For the control group, the same volume of PBS was injected into the intervertebral disc. Intervertebral disc tissues were collected 4 weeks post‐surgery for subsequent experimental analyses [35].

### 2.19 Immunofluorescence Staining

For in situ detection of protein expression, dewaxed human and rat tissue sections were placed in citrate buffer (pH 6.5) and heated at 65°C for 3 h for antigen retrieval. Sections were blocked with PBS containing 5% bovine serum albumin at 37°C for 1.5 h and then incubated with primary antibodies overnight at 4°C (Immunoway, Product Nos. YM8147 and YN0996). After three washes with PBS containing 0.5% Tween‐20, sections were incubated with secondary antibodies (Servicebio, GB22301 and GB21303) at 37°C for 1 h. When co‐labelling with apoptotic cells was required, TUNEL staining was performed after secondary antibody incubation. Sections were mounted with DAPI‐containing mounting medium (Servicebio, G1407) and imaged using a fluorescence microscope (Olympus BX53). Positive staining areas and positive‐cell proportions were quantified using ImageJ.

### 2.20 Histological Analysis of Human and Rat Intervertebral Disc Tissues

Human and rat NP tissues were fixed in 4% paraformaldehyde immediately after isolation, embedded in paraffin and sectioned. For rat samples, the entire intervertebral disc was harvested, fixed, and embedded using the same protocol. Five‐micrometre paraffin sections were stained with haematoxylin and eosin (H&E; Servicebio, G1053) to evaluate the overall disc structure, including the AF, NP and cartilaginous endplate. Safranin O‐fast green (SOFG; Servicebio, G1005) staining was used to visualise proteoglycan and cartilage content and to assess disc degeneration under different interventions. H&E‐ and SOFG‐stained sections were examined under an optical microscope (Leica DM3000), and collagen heterogeneity was assessed under polarised light.

### 2.21 Isolation and Flow Cytometric Sorting of Disc‐Derived Macrophages

To obtain disc‐derived macrophages for ex vivo culture and cytological analyses, intervertebral disc tissues were harvested from rats in each experimental group 4 weeks after surgery. After euthanasia, the caudal intervertebral discs were aseptically isolated, and surrounding soft tissues were carefully removed under a stereomicroscope. The collected disc tissues were washed three times with cold phosphate‐buffered saline (PBS) containing 1% penicillin–streptomycin and then minced into small fragments using sterile ophthalmic scissors. The minced tissues were enzymatically digested in serum‐free DMEM/F12 containing 0.2% type II collagenase, 2 U/mL dispase II, and 100 U/mL DNase I at 37°C for 60–90 min with gentle agitation. The cell suspension was gently pipetted every 15 min to facilitate tissue dissociation. After digestion, the suspension was filtered sequentially through 70 and 40‐μm cell strainers to remove undigested tissue debris. Cells were then centrifuged at 400 × *g* for 5 min at 4°C, resuspended in red blood cell lysis buffer for 2–3 min when necessary, and washed twice with fluorescence‐activated cell sorting (FACS) buffer consisting of PBS supplemented with 2% fetal bovine serum (FBS) and 1 mM EDTA.

For flow cytometric sorting, single‐cell suspensions were incubated with Fc receptor‐blocking reagent for 10 min at 4°C to reduce nonspecific antibody binding. Cells were then stained with fluorophore‐conjugated antibodies against anti‐rat CD45‐APC‐Cy7 (202216, BioLegend), anti‐rat CD3 APC (201414, BioLegend), anti‐rat CD45R‐FITC (11‐0460‐82, Invitrogen), anti‐rat CD11b/c‐BV421 (743977, BD Biosciences), and anti‐rat CD163‐PE (MCA342PE, Bio‐Rad) for 30 min at 4°C in the dark. A fixable viability dye was used to exclude dead cells. Unstained controls and fluorescence minus one control were included to determine gating boundaries. The gating strategy was performed as follows. Cellular debris was first excluded based on forward scatter and side scatter properties. Single cells were then selected using FSC‐A/FSC‐H and SSC‐A/SSC‐H parameters, followed by exclusion of dead cells using the viability dye. CD45^+^ leukocytes were subsequently gated, and CD3^+^ T cells and CD45R^+^ B cells were excluded. Finally, disc‐derived macrophage‐enriched cells were identified and sorted as live CD45^+^CD3^−^CD45R^−^CD11b/c^+^CD163^+^ cells using a fluorescence‐activated cell sorter.

Freshly sorted cells were collected into complete culture medium consisting of DMEM/F12 supplemented with 10% FBS, 1% penicillin–streptomycin, and 20 ng/mL macrophage colony‐stimulating factor. For gene expression analysis, freshly sorted cells were immediately lysed for RNA extraction, followed by qRT‐PCR analysis of senescence‐, inflammation‐, lipid metabolism‐, and efferocytosis‐related genes, including *Cdkn1a, Cdkn2a, Il1b, Pparg, Abca1, Abcg1* and *Mertk*. For cytological assays, sorted cells were seeded onto sterile glass coverslips or culture plates and maintained at 37°C in a humidified incubator with 5% CO_2_. After attachment, cells were subjected to immunofluorescence staining or functional assays to evaluate macrophage polarisation and cellular phenotypes.

### 2.22 Statistical Analysis

All quantitative data in this study are presented as the mean ± standard deviation (SD). Biological replicate numbers are indicated in the corresponding figure legends. In vitro experiments were performed with at least three independent biological replicates, and technical replicates were included for qRT‐PCR where appropriate. Differences between two independent groups were analysed using a two‐tailed unpaired Student's *t*‐test. For comparisons among three or more groups, one‐way analysis of variance (ANOVA) was performed, followed by appropriate post hoc multiple‐comparison tests when necessary. Correlation analyses were conducted using Pearson's correlation coefficient. Statistical significance was defined as *p* < 0.05. All statistical analyses were performed using R software (v4.3.1) and GraphPad Prism 9.0.

## 3 Results

### 3.1 IVDD is Accompanied by Lipid Metabolism Disorder

**FIGURE 1:**
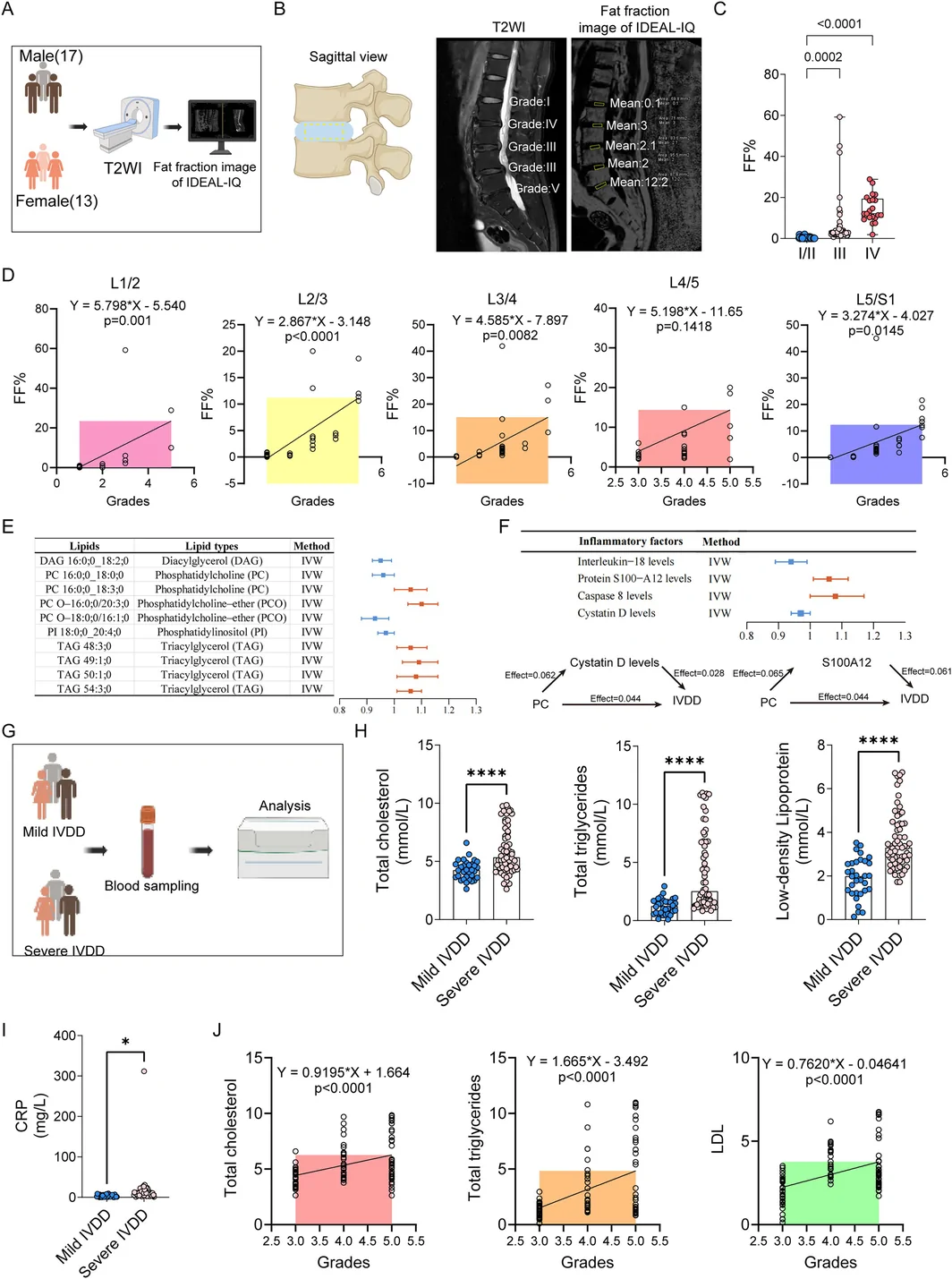
Intervertebral disc degeneration accompanied by lipid metabolism disorder. (A) Schematic diagram of T2WI imaging using the IDEAL‐IQ sequence in 17 male and 13 female subjects; (B) T2WI images of the IDEAL‐IQ sequence showing the distribution of fat fraction in intervertebral discs with different degeneration grades (Grade I–V); (C) box plot of degeneration grades and fat fraction; (D) fitting curve of degeneration grades and fat fraction in different lumbar segments; (E) Mendelian randomisation analysis of intervertebral disc degeneration and key lipid types (IVW method); (F) Mendelian mediation analysis of key inflammatory factors between lipids and IVDD; (G) schematic diagram of the peripheral blood study in clinical patients; (H) bar chart of total cholesterol, triglycerides, and low‐density lipoprotein in patients with mild/severe IVDD; (I) bar chart of CRP in patients with mild/severe IVDD; (J) bar chart of total cholesterol, triglycerides, and low‐density lipoprotein in intervertebral discs with different degeneration grades. *p < 0.05, **p < 0.01, ***p < 0.001, ****p < 0.0001.

To investigate the association between lipid metabolism and the degree of intervertebral disc degeneration (IVDD), we enrolled 30 subjects (17 males and 13 females). The relationship between lumbar disc status and fat fraction (FF) was evaluated using T2‐weighted imaging (T2WI) and IDEAL‐IQ fat fraction imaging (Figure 1A, Table S1). Figure 1B shows the measurement regions of disc FF (the regions of interest are indicated by the yellow dashed box in the scanned image). As illustrated, analysis of the sagittal lumbar disc region revealed significant differences in FF among discs of different degeneration grades (Grades I–V). Specifically, the mean FF of Grade V discs reached 12.2%, which was substantially higher than that of Grades I–III (ranging from 0.1% to 3.0%) (Figure 1B). Comprehensive analysis of FF data from all lumbar discs of the 30 enrolled subjects demonstrated a significant upward trend in FF as the degeneration grade increased (Figure 1C). Further correlation analyses were performed between FF and degeneration grade for each disc segment (L1/2 to L5/S1). The results indicated that FF was positively correlated with degeneration grade in the L1/2, L2/3, L3/4, and L5/S1 segments, while no significant correlation was observed in the L4/5 segment (Figure 1D, Table S1). To further elucidate the pathogenic effect of lipid homeostasis imbalance on IVDD, we conducted a Mendelian randomisation analysis using the FinnGen database. Mendelian randomisation results suggested that multiple lipids are associated with IVDD, including diacylglycerols, phosphatidylcholines and triglycerides. Moreover, these lipid molecules may mediate the formation of the local inflammatory microenvironment in the intervertebral disc through inflammatory factors such as cystatin D and S100A12 protein (Figure 1E,F). In addition, we analysed blood samples from IVDD patients (all with normal BMI) collected in our institution, stratified by mild/severe IVDD (Figure 1G, Table S2). The results showed that the levels of total cholesterol, triglycerides, and low‐density lipoprotein (LDL) in the severe IVDD group were significantly higher than those in the mild IVDD group (Figure 1H). Meanwhile, the level of C‐reactive protein (CRP) was also significantly elevated in the severe IVDD group (Figure 1I). Further correlation analysis confirmed that total cholesterol, triglyceride, and LDL levels were all positively correlated with the grade of intervertebral disc degeneration (Figure 1J).

Together, clinical imaging, Mendelian randomisation, and patient blood analyses support a close association between lipid metabolic disorder, inflammatory activation and IVDD progression.

### 3.2 Lipid Metabolism‐Related Genes Define Clinically Relevant Molecular Subtypes of IVDD

**FIGURE 2:**
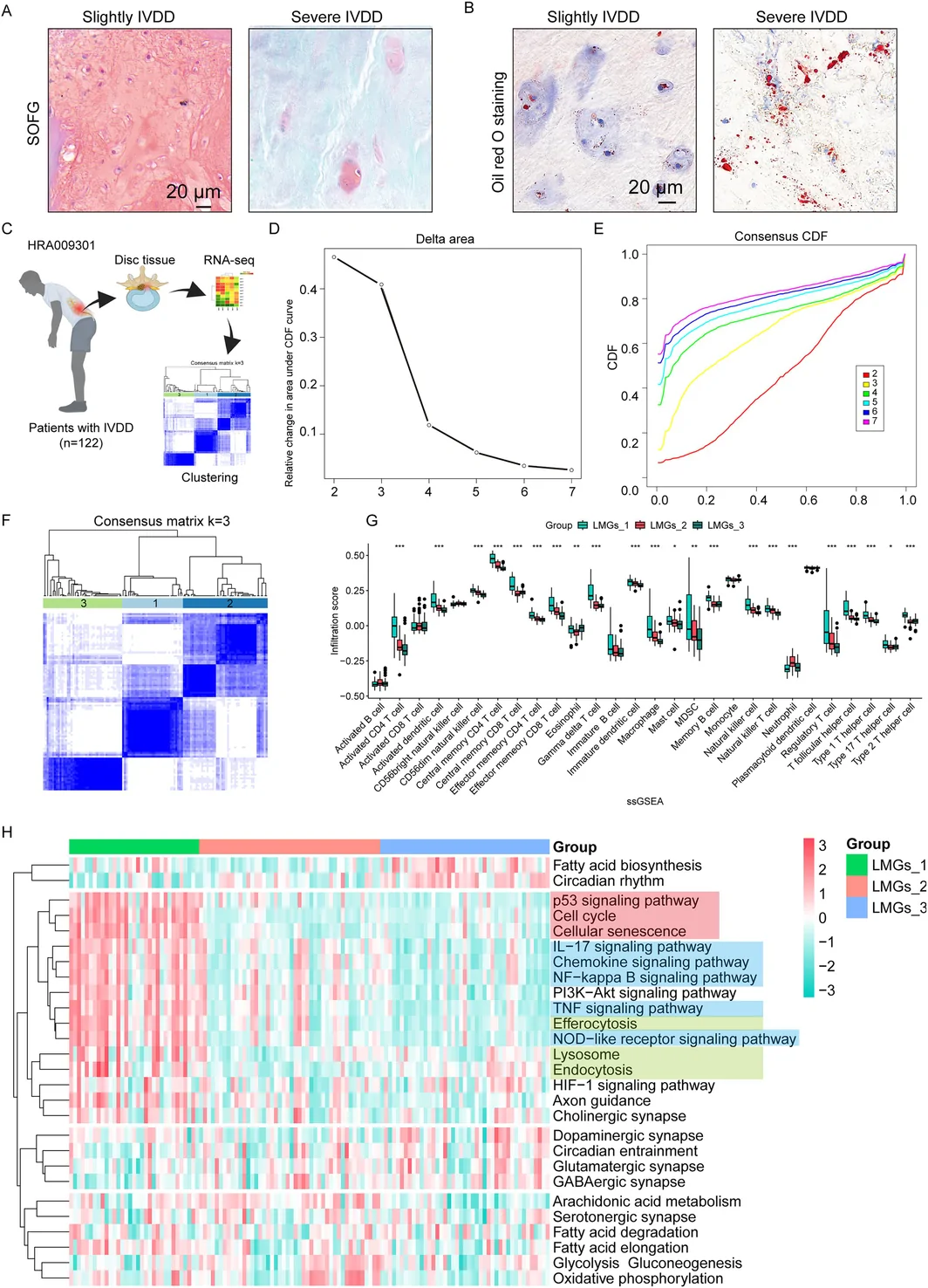
Establishing clinical‐molecular classification of lumbar intervertebral disc degenerative diseases based on lipid metabolism gene set. (A) SOFG staining of mild IVDD and severe IVDD; (B) oil red staining of mild IVDD and severe IVDD; (C) schematic diagram of clustering analysis of 122 patients with lumbar disc herniation (RNA sequencing data from the HRA009301 dataset) using the ConsensusClusterPlus method; (D) delta area curve analysis of consensus clustering to determine the optimal number of clusters; (E) consensus CDF curve of bulk transcriptomic sample; (F) consensus matrix heatmap of sequencing data from 122 IVDD patients when *k* = 3; (G) box plot of infiltration levels of three subgroups (LMGs_1, LMGs_2, LMGs_3) with different immune cells (such as macrophages, T cells, NK cells, etc.); (H) violin plot of representative signalling pathways in the three subgroups.

Building on the above results, we collected intervertebral disc tissues with different degrees of degeneration for histological studies. SOFG staining and Oil Red O staining of intervertebral disc tissues showed less lipid deposition (red‐stained areas) in mild IVDD samples, whereas the red‐stained areas were significantly increased and widely distributed in severe IVDD samples (Table S3). These findings indicate that the level of lipid deposition increases with the severity of IVDD, and lipid metabolism disorder is one of the core pathological features of IVDD (Figure 2A,B). Subsequently, we analysed the transcriptome sequencing data of intervertebral disc tissue samples from 122 publicly available IVDD patients (HRA009301) to clarify the close correlation between lipid homeostasis and IVDD at the molecular level (Figure 2C). The consensus clustering algorithm showed that as the number of clusters *k* increased from 2 to 7, the relative change in the area under the CDF curve decreased significantly and tended to be flat when *k* = 3, indicating that *k* = 3 was the optimal number of clusters. The matrix showed obvious block characteristics, and the consensus coefficient (colour depth) of samples in the same cluster was higher, verifying the reliability of the clustering result when *k* = 3. Finally, the samples were divided into 3 molecular subtypes (LMGs_1, LMGs_2, LMGs_3) (Figure 2D–F). Given the important promoting role of inflammatory activation in IVDD, we first performed ssGSEA‐based immune cell infiltration scoring. The results indicated that the LMGs_1, LMGs_2 and LMGs_3 subtypes had significant differences in the infiltration scores of various immune cells, suggesting that the immune microenvironment of different molecular subtypes is heterogeneous (Figure 2G). Notably, the LMGs_1 subtype had the highest inflammation score, indicating that it may be the most critical IVDD‐promoting subtype (Figure 2G).

To further explore the functional differences among the subtypes, we conducted KEGG enrichment analysis. The KEGG pathway enrichment heatmap showed that the core functional pathways of the 3 molecular subtypes were biased. The LMGs_1 subtype was enriched in cell homeostasis regulatory pathways such as ‘cell senescence’ and ‘p53 signalling pathway’, as well as inflammatory pathways such as ‘IL‐17 signalling pathway’ and ‘NF‐κB signalling pathway’; the LMGs_2 subtype was highly enriched in pathways such as arachidonic acid metabolism, fatty acid degradation, fatty acid elongation, oxidative phosphorylation, and glycolysis; the LMGs_3 subtype was enriched in metabolic pathways such as ‘fatty acid biosynthesis’ and ‘circadian rhythm’ (Figure 2H). Therefore, the establishment of clinical‐molecular classification of lumbar disc degenerative disease based on lipid metabolism gene sets indicates that lipid metabolism patterns are closely related to the immune senescence microenvironment of intervertebral discs, and the LMGs_1 subtype may be the most critical pathological subtype of IVDD.

### 3.3 Transcriptomic Profiling Links IVDD to Lipid Metabolic Disorder, Cellular Senescence, and Phagocytosis‐Related Pathways

**FIGURE 3:**
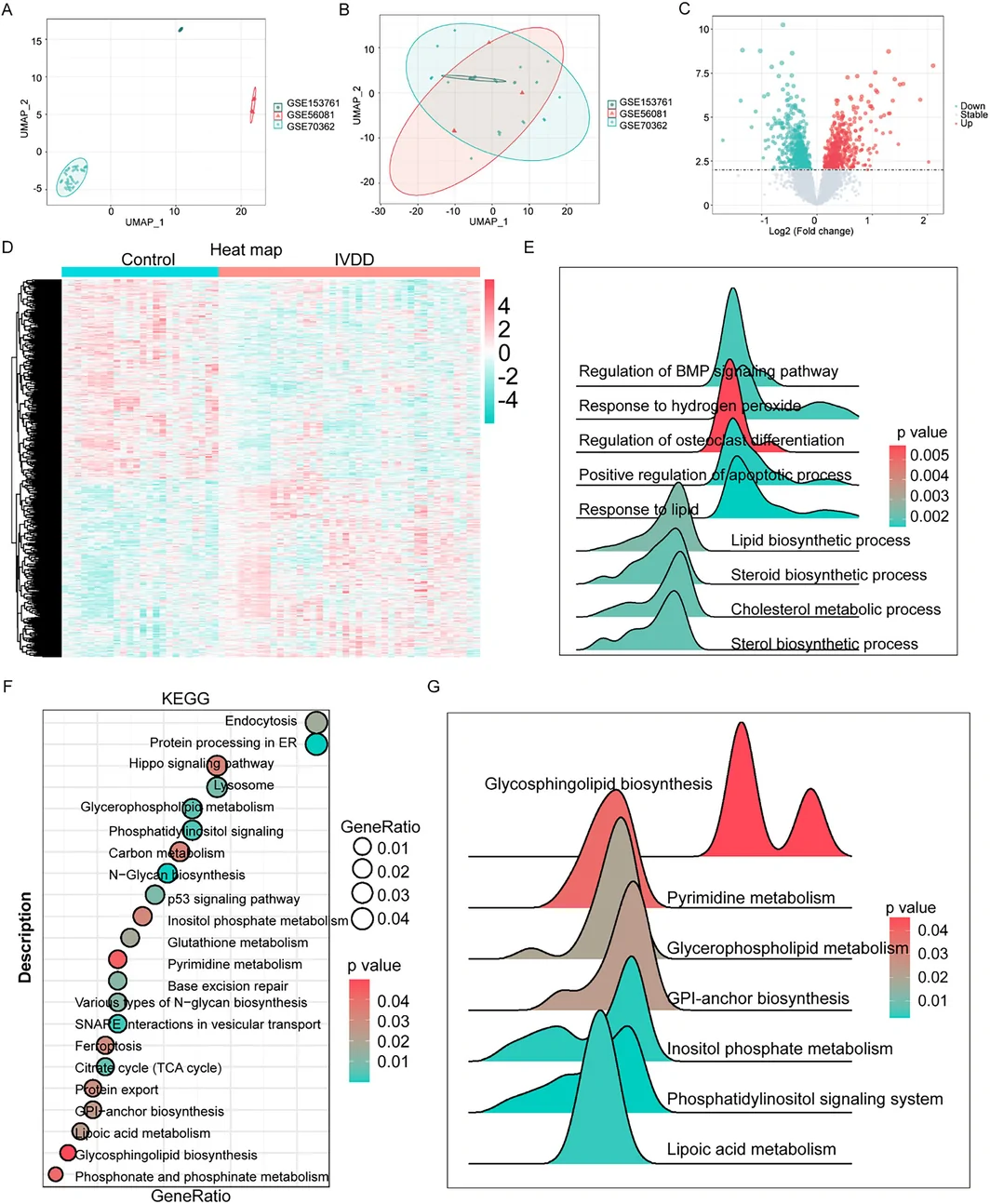
Transcriptome sequencing reveals that intervertebral disc degeneration is accompanied by lipid metabolism disorders, changes in cell senescence and cell phagocytosis‐related functions. (A) Principal component analysis (PCA) of three original transcriptome datasets, GSE56081, GSE70362, and GSE153761‐without batch effect removal; (B) principal component analysis (PCA) of three original transcriptome datasets with batch effect removal; (C) volcano plot of differential genes between the control group and IVDD group after integrating three transcriptome sequencing datasets; (D) heatmap of differential genes between the control group and IVDD group after integrating three transcriptome sequencing datasets; (E) GSEA plot of lipid metabolism‐related differential genes between the control group and IVDD group after integrating three transcriptome sequencing datasets; (F) KEGG enrichment plot of differential genes between the control group and IVDD group after integrating three transcriptome sequencing datasets; (G) GSEA plot of differential signalling pathways between the control group and IVDD group after integrating three transcriptome sequencing datasets.

To further explore the predictive value of lipid homeostasis for IVDD and the underlying regulatory mechanisms, we included samples from 3 public datasets for analysis (GSE153761, GSE56081 and GSE70362). Figure 3A shows the PCA plot of samples from the three datasets before batch effect correction (Figure 3A), whereas (Figure 3B) shows the PCA plot after batch effect correction (Figure 3B). The integrated data were divided into two groups: the Control group and the IVDD group, and the volcano plot of differential genes between them is shown in the figure (Figure 3C). Figure 3D displays the heatmap of differential genes, including 523 upregulated genes and 587 downregulated genes (Figure 3D). Subsequently, we performed GSEA analysis on key GO pathways. The results indicated that intervertebral disc degeneration is accompanied by changes in multiple key pathways, including the activated BMP signalling pathway, hydrogen peroxide signalling pathway, osteoclast differentiation and cell apoptosis. Meanwhile, lipid metabolism‐related pathways were significantly inhibited, including lipid biosynthetic process, steroid biosynthetic process and cholesterol metabolism (Figure 3E). Both KEGG pathway enrichment results and GSEA showed that differential genes were enriched in ‘endocytosis, p53 pathway, various lipid metabolism‐related pathways’ and other pathways, all of which exhibited significant differences (Figure 3F,G). These results suggest that lipid metabolic disorder, cellular senescence, and altered phagocytic activity are key pathological features of IVDD.

### 3.4 Lipid Metabolism‐Related Hub Genes Are Associated With IVDD Progression Risk

We next used machine learning to identify lipid metabolism‐related genes with predictive value for IVDD. Intersecting transcriptomic DEGs with LMGs yielded 63 candidate genes (Figure S1). LASSO regression analysis was performed on these 63 genes, leading to the identification of 19 hub genes (Figure S1B,C). Furthermore, ROC analysis of the initial LASSO model in the discovery dataset showed an AUC of 1.000 (Figure S1D). Because this exceptionally high value may reflect optimistic performance estimation or potential overfitting, this model was used primarily as a feature‐screening step rather than as a standalone diagnostic classifier. Moreover, the risk score for IVDD was found to be significantly higher than that of the control group, suggesting that these genes have potential as diagnostic markers for IVDD (Figure S1E). Subsequently, we used three machine learning algorithms, CatBoost, LightGBM, and XGBoost, to analyse the value of hub genes in predicting IVDD. The SHAP methodology was used to interpret the three ML models, yielding Summary Plots and Beeswarm Plots. The Summary Plots of the three machine learning algorithms showed the predictive value ranking of the 19 genes; for example, TIAM2, MTMR2, PPT1, SLC44A4, and GPD2 exhibited relatively high predictive value (Figure S1F–H). Beeswarm Plots further indicated that GPD2 was positively correlated with IVDD risk, while TIAM2, MTMR2, SLC44A4, and PPT1 were negatively correlated with IVDD risk (Figure S1I–K).

**FIGURE 4:**
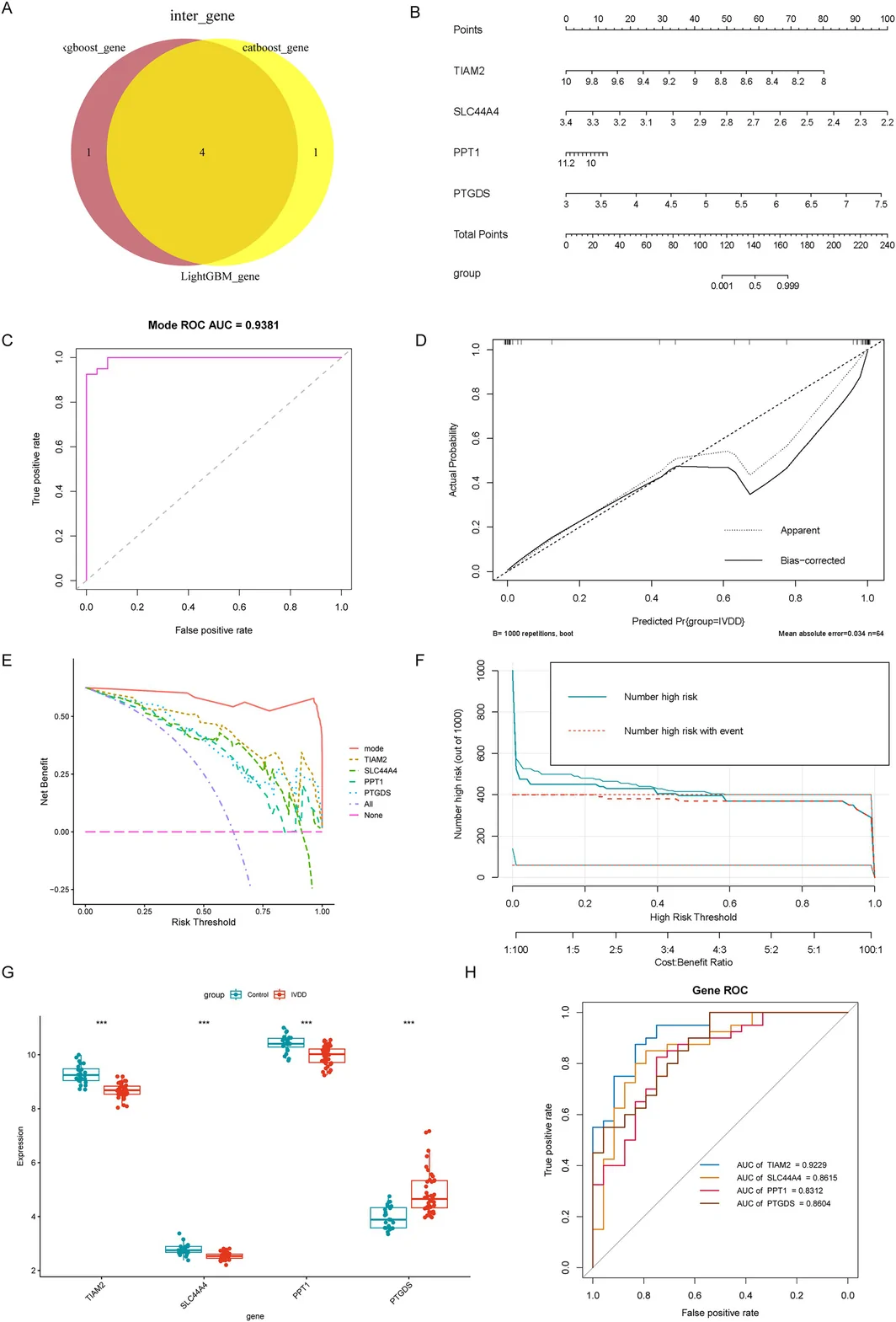
The lipid metabolism hub genes related to IVDD can accurately predict the risk of IVDD progression. (A) The top 10 genes from Catboost, LightGBM, and Xgboost were further intersected to obtain 4 hub genes; (B) nomogram of the 4 hub genes; (C) ROC curve of the Nomogram model; (D) calibration curve predicted by the Nomogram model; (E) DCA curve predicted by the Nomogram model; (F) performance of the Nomogram in the training set (integrated dataset) of IVDD; (G) expression results of the 4 hub genes in normal and degenerative tissue transcriptome sequencing; (H) ROC curves of the 4 hub genes.

Therefore, based on the above results, we further screened 4 common differential genes (TIAM2, SLC44A4, PPT1, PTGDS) from the top 5 hub genes of the three machine learning algorithms, which were used as the core variables of the prediction model (Figure 4A). A nomogram was further constructed based on these 4 genes; the expression level of each gene corresponds to a different score, and the total score is obtained by summing the scores of each gene. The individual's disease risk probability is then converted from the total score, providing a potentially interpretable framework for individualised IVDD risk estimation pending further external validation (Figure 4B). The AUC of the ROC curve of the model was 0.9381, indicating that the model has good predictive value (Figure 4C). The calibration curve illustrated excellent concordance between predicted and observed outcomes (Figure 4D). Furthermore, the DCA curve and clinical impact curves (CIC) indicated the strong performance of the risk model (Figure 4E,F). In the established model, the expression of PTGDS was significantly elevated in the IVDD group, whereas the expression of TIAM2, SLC44A4, and PPT1 was notably lower than those in the control group (Figure 4G). The ROC curves for these hub genes exhibited AUC values of 0.9229, 0.8615, 0.8312, and 0.8604, respectively, underscoring their potential as valuable biomarkers for IVDD (Figure 4H).

Together, these analyses indicate that lipid metabolism‐related hub genes can stratify IVDD progression risk and may serve as candidate biomarkers.

### 3.5 Lipid Metabolism‐Related Hub Genes Are Closely Linked to the Disc Immune Microenvironment

**FIGURE 5:**
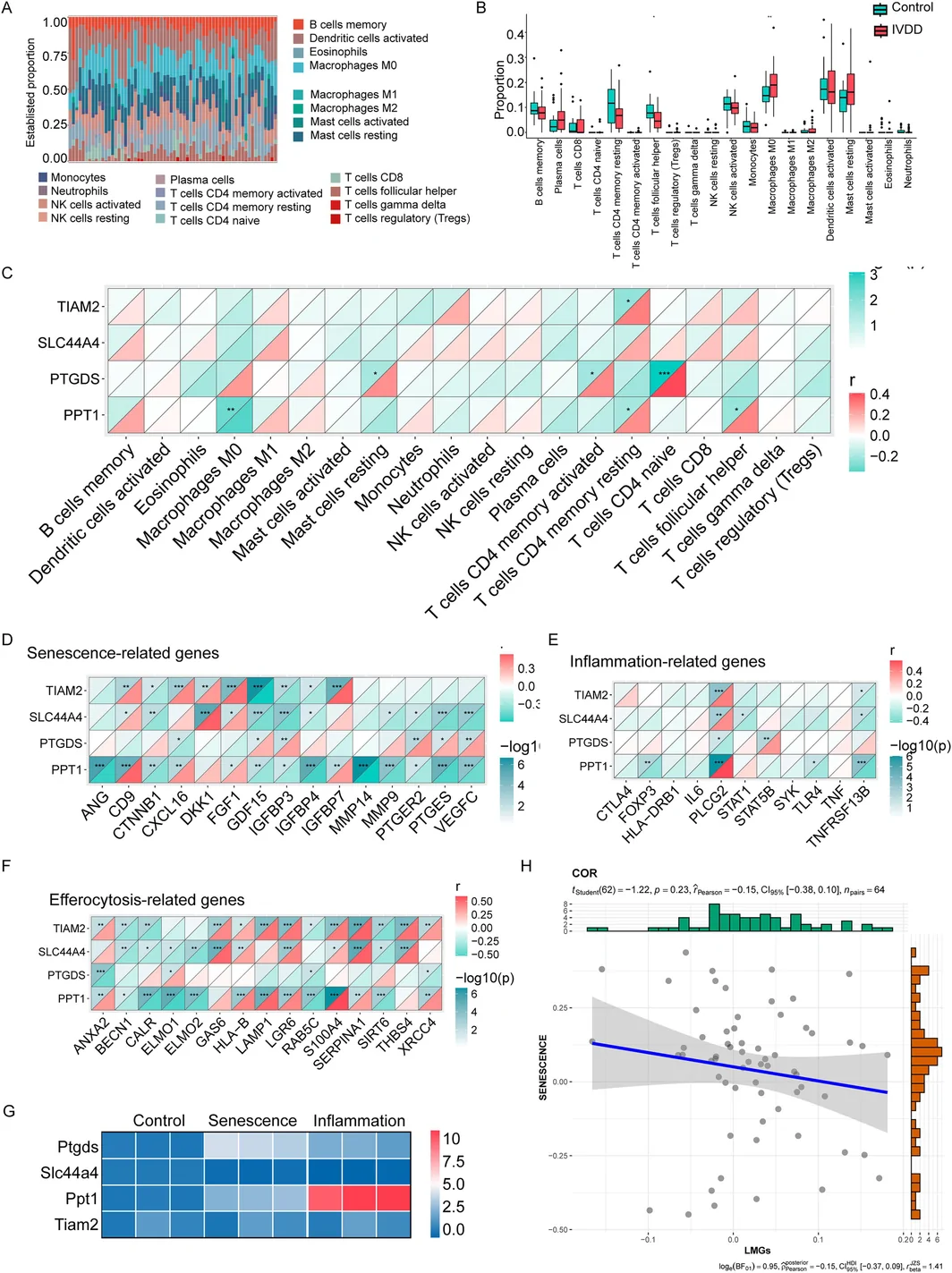
The expression of lipid metabolism hub genes is closely related to the immune microenvironment of the intervertebral disc. (A) Bar chart showing the distribution of 20 different immune cells in various IVDD samples; (B) box plot illustrating the expression profiles of 20 immune cells in the IVDD group and control group; (C) correlation diagram of the 4 hub genes with 20 different immune cells; (D) correlation diagram of hub genes with cellular senescence; (E) correlation diagram of hub genes with inflammation; (F) correlation diagram of hub genes with efferocytosis; (G) the results of the hub gene expression in senescent and inflammatory macrophages, respectively (*n* = 3). (H) Correlation diagram of lipid metabolism‐related genes and cellular senescence‐related genes.

To examine the relationship between hub genes and immune cell infiltration, we applied CIBERSORT. Figure 5A shows the distribution of 20 immune cell types across samples, and Figure 5B compares immune cell abundance between control and IVDD tissues. Among these cell types, M0 macrophages were significantly increased in IVDD, suggesting a close relationship between lipid metabolism‐related hub genes and macrophage infiltration in the disc microenvironment. We then mapped core gene expression across immune cell subsets, including B cells, dendritic cells, macrophage subtypes, and T cell subsets, further indicating that these genes are involved in immune regulation (Figure 5C). KEGG enrichment analysis suggested that PPT1 is mainly associated with lysosomal function and autophagy; PTGDS with reactive oxygen species, autophagy, and oxidative phosphorylation; SLC44A4 with tight junctions, autophagy, and glycolysis; and TIAM2 with PI3K‐AKT signalling, mitophagy and efferocytosis (Figure S2A,D). These findings suggest that lipid metabolism‐related hub genes converge on autophagy, macrophage efferocytosis, and inflammatory regulation during IVDD progression.

To explore the association patterns of core genes with genes related to senescence, inflammation, and efferocytosis, we performed heatmap visualisation of genes affected by core genes, respectively. Figure 5D presents the correlation and significance between core genes and senescence‐related genes such as ANG, CD9, and CXCL16, with the colour gradient reflecting both the correlation coefficient (*r*) and statistical significance (Figure 5D). The results showed that core genes were significantly associated with multiple senescence‐related genes (e.g., PPT1 was significantly correlated with ANG, CD9, etc.). In the inflammatory gene correlation analysis, Figure 5E showed that some core genes were significantly associated with inflammatory regulatory genes such as PTGDS, IL6 and PLCG2 (Figure 5E). In the efferocytosis gene correlation analysis, Figure 5F showed that core genes (e.g., TIAM2) were significantly associated with efferocytosis‐related genes such as ANXA2 and BECN1 (Figure 5F). Given the critical role of macrophages in IVDD microenvironment, we established senescent (Dox, 100 nM) or inflammatory (LPS, 10 μg/mL) macrophage model [36, 37]. The results of qRT‐PCR demonstrated consistent tendency of the hub genes with the results of bulk RNA sequencing (Figure 5G). Notably, scatter plot and COR analysis showed that cellular senescence was weakly negatively correlated with lipid metabolism genes (*τ* = −0.15, *p* = 0.23), with a 95% confidence interval of [−0.38, 0.10] (Figure 5H). This result suggests that there is a certain negative correlation between the senescence process and lipid metabolism molecular characteristics in IVDD, and core genes, as the intersection node between the two, may be involved in balancing senescence and tissue homeostasis.

These results link lipid metabolism‐related hub genes to immune remodelling in IVDD and suggest that autophagy, efferocytosis, and senescence are key components of this regulatory network.

### 3.6 Single‐Cell Analysis Reveals Lipid Metabolism‐Dependent Regulation of Macrophage Senescence and Inflammatory States in Human IVDD

**FIGURE 6:**
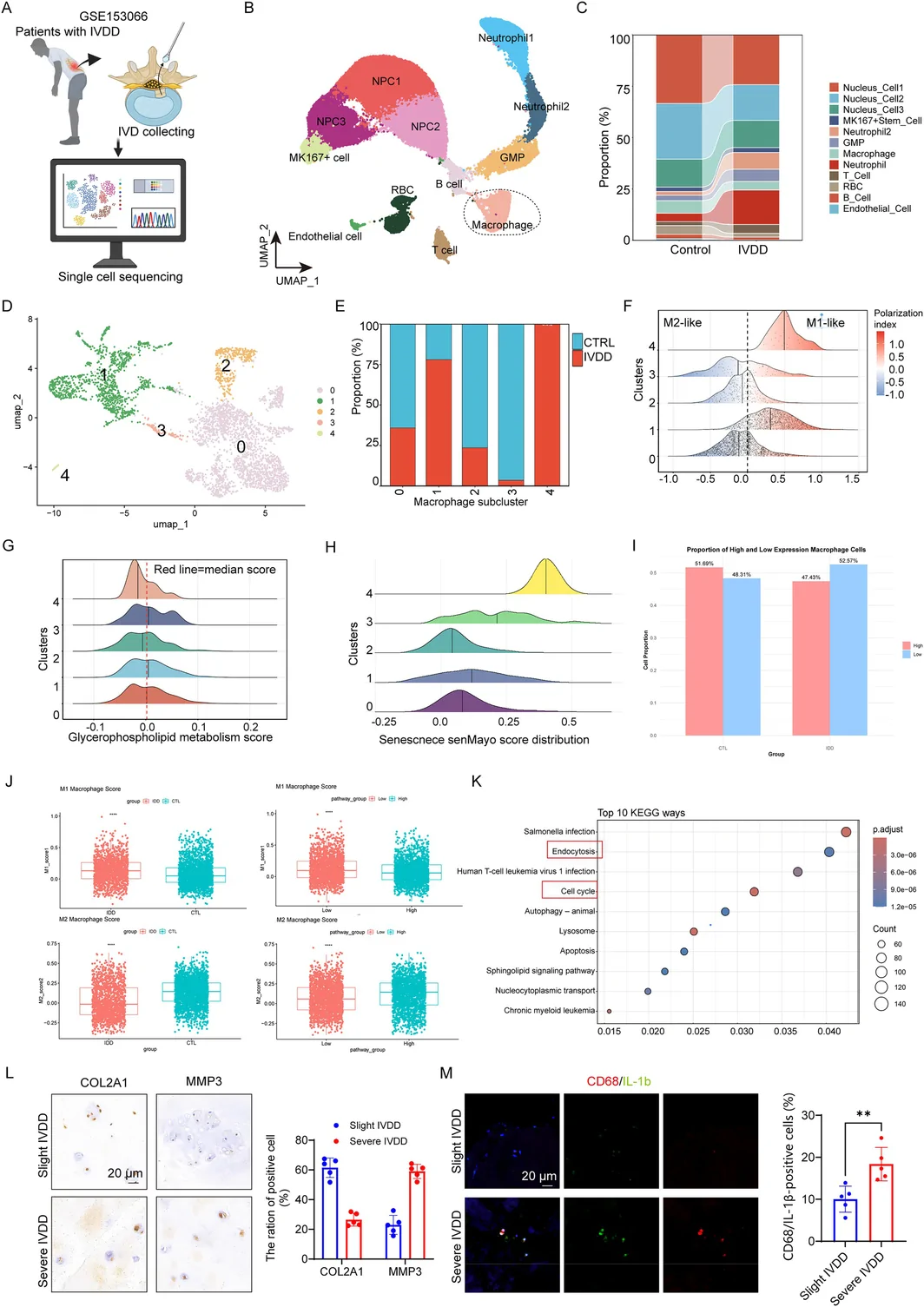
Single‐cell sequencing of human intervertebral discs reveals that lipid metabolism disorders are involved in the regulation of macrophage senescence and inflammatory phenotypes. (A) Flow chart of the single‐cell sequencing experiment; (B) UMAP plot of scRNA‐seq; (C) proportion plot of intervertebral disc cell subgroups; (D) UMAP clustering plot of macrophage subgroups; (E) proportion plot of macrophage subgroups in the IVDD and control groups; (F) M1 and M2 polarisation scores of intervertebral disc macrophage subgroups; (G) lipid metabolism score levels of intervertebral disc macrophage subgroups; (H) senescence score levels of intervertebral disc macrophage subgroups; (I) proportion plot of macrophages grouped by lipid metabolism score in CTRL and IVDD, respectively; (J) box plot of M1 and M2 scores of macrophages grouped by lipid metabolism score in CTRL and IVDD, respectively; (K) enrichment pathway plot of differentially expressed genes between the two groups of macrophages grouped by lipid metabolism score; (L) immunohistochemical staining of intervertebral disc tissues from patients with mild and severe IVDD (MMP3, COL2A1, 20 μm); (M) immunofluorescence images of intervertebral disc tissues from patients with mild and severe IVDD (CD68/IL‐1b). *p < 0.05, **p < 0.01, ***p < 0.001, ****p < 0.0001.

To define how macrophage lipid homeostasis shapes the IVDD immune microenvironment, we re‐analysed publicly available scRNA‐seq data from human IVDD samples (GSE153066) (Figure 6A). UMAP results indicated that we identified a total of 12 major cell clusters, and the expression profiles of representative markers for each cell type are shown in the dot plot (Figure 6B). The marker genes of each cell subset are displayed in the bubble plot (Figure S3A). The stacked bar chart of cell type proportions showed significant differences in cell composition between the control group and the IVDD group: the proportions of immune cells such as macrophages and neutrophils in the IVDD group were significantly higher than those in the control group, while the proportion of nucleus pulposus cell subsets was relatively decreased. These results suggest that IVDD progression is accompanied by immune cell infiltration and remodelling of intrinsic cell proportions, which is one of the core features of pathological microenvironment changes in IVDD (Figure 6C). Combined with previous data and existing literature, macrophage infiltration is the core cellular mechanism in the formation of the inflammatory microenvironment of IVDD. Therefore, we focused on lipid homeostasis and macrophage function regulation. After secondary clustering of macrophages, the UMAP plot (Figure 6D) divided them into 5 functional subsets (Cluster 0–4), and each subset exhibited independent clustering characteristics in the UMAP space (Figure 6D). The bar chart (Figure 6E) showed the proportional differences of each macrophage subset between the CTL and IVDD groups, reflecting the functional heterogeneity of macrophages in IVDD tissues (Figures 6E and S3B). First, we analysed the polarisation index of each macrophage subset. The violin plot of polarisation index (Figure 6F) showed significant differences in polarisation status among different macrophage subsets: Cluster 4 tended to be M1‐type (pro‐inflammatory) polarised, while Cluster 0 was more inclined to be M2‐type (anti‐inflammatory) polarised (Figure 6F). Given that Cluster 4 was mainly present in the IVDD group, it indicates that the polarisation of macrophages to the pro‐inflammatory phenotype is an important immune regulatory feature during IVDD progression (Figure 6E). The ridgeline plot showed the distribution characteristics of lipid metabolism scores among each macrophage subset: there were significant differences in lipid metabolism scores among different subsets, and the lipid metabolism score of Cluster 0 was significantly higher than that of Cluster 4 (Figure 6G). The ridgeline plot of senescence scores showed that the senescence score of Cluster 4 was significantly higher than that of Cluster 0 (Figure 6H). The above results suggest that lipid metabolic reprogramming is involved in the regulation of the inflammatory characteristics and senescent phenotype of macrophages.

To further explore the key role of macrophage lipid homeostasis in regulating the inflammatory characteristics and senescent phenotype of macrophages, we divided macrophages into two groups (low and high lipid metabolism) based on their lipid metabolism scores. The bar chart of cell proportions showed that in the IVDD group, the proportion of macrophages with high lipid metabolism scores was significantly lower than that of macrophages with low lipid metabolism scores, while in the CTRL group, the proportion of macrophages with high lipid metabolism scores was significantly higher than that of macrophages with low lipid metabolism scores (Figure 6I). Meanwhile, we performed polarisation scoring on the two groups of macrophages with different lipid metabolism scores, respectively. The results indicated that macrophages with high lipid metabolism scores tended to have an M2 phenotype, while macrophages with low lipid metabolism scores were more inclined to have an M1 phenotype (Figure 6J). KEGG pathway enrichment results of differential genes between the two groups of macrophages showed that the differential genes were significantly enriched in pathways such as Endocytosis, Cell cycle and Lysosome (Figure 6K). These results indicate that low lipid metabolic activity pushes macrophages into an early differentiation state typified by pro‐inflammation, cellular senescence and defective efferocytosis. Conversely, high lipid metabolic activity drives maturation toward a late‐stage anti‐inflammatory homeostatic phenotype that maintains normal efferocytosis function.

We then validated these single‐cell findings in human intervertebral disc tissues with different degeneration severities. Immunohistochemistry showed that mildly degenerated samples contained more COL2A1‐positive matrix‐synthesising cells and fewer MMP3‐positive matrix‐degrading cells than severely degenerated samples (Figure 6L). Immunofluorescence double labelling showed a higher proportion of CD68/IL‐1β double‐positive cells in severe IVDD tissues than in mild IVDD tissues (*p* < 0.01), indicating increased pro‐inflammatory macrophage infiltration (Figure 6M). Together with transcriptomic analyses, these histological findings support a close association between macrophage lipid metabolic disorder, senescence, and inflammatory activation during IVDD progression.

### 3.7 Macrophage Lipid Dysregulation Promotes Senescence‐Dependent Impairment of Efferocytosis

**FIGURE 7:**
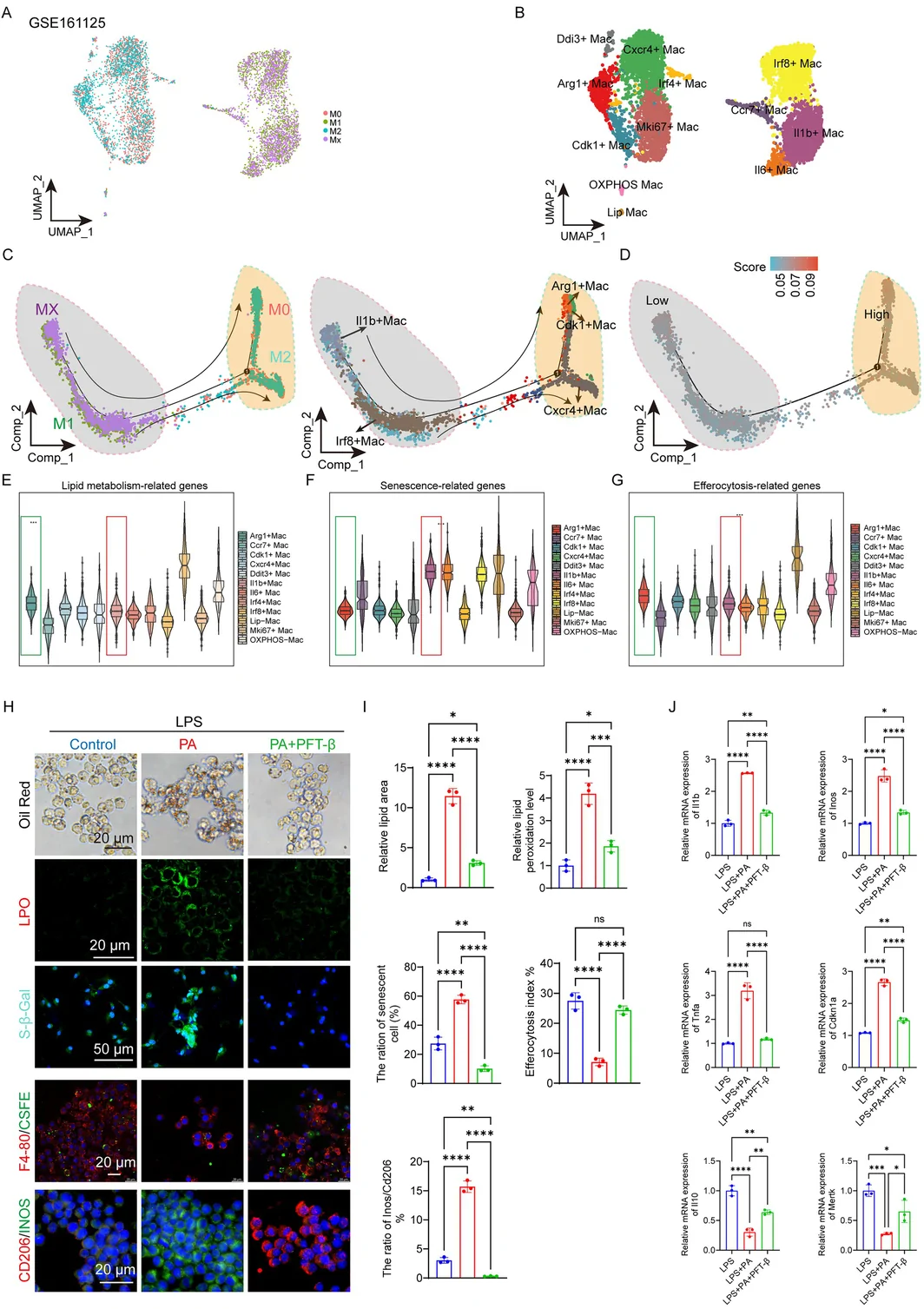
Pro‐inflammatory macrophages are accompanied by lipid metabolism disorders, cellular senescence and impaired efferocytosis. (A) UMAP plot of 4 cell samples from the public database (GSE161125); (B) UMAP clustering plot of macrophage subgroups (including Arg1+, Il1b+, Cxcr4+ and other subgroups); (C) time series analysis results of each sample and macrophage subgroup; (D) dynamic changes of lipid metabolism scores of cell subgroups in the time series; (E) lipid metabolism scores of different macrophage subgroups; (F) senescence scores of different macrophage subgroups; (G) efferocytosis scores of different macrophage subgroups; (H, I) results of oil red staining, lipid peroxidation staining, senescence staining, autophagy detection, polarisation staining and their quantification (*n* = 3); (J) expression levels of genes related to senescence, inflammation and efferocytosis in macrophages of each group (*n* = 3). *p < 0.05, **p < 0.01, ***p < 0.001, ****p < 0.0001.

To investigate how lipid homeostasis regulates macrophage function, we re‐analysed a public macrophage polarisation dataset (GSE161125). According to treatment conditions, macrophages were classified into four functional phenotypes: M0 (resting), M1 (pro‐inflammatory), M2 (anti‐inflammatory) and Mx (transitional) (Figure 7A). UMAP re‐clustering identified 10 macrophage subclusters (Figures 7B and S4A,B). Monocle2 pseudotime analysis showed that M2 macrophages were mainly distributed at late differentiation stages, whereas M1 macrophages were enriched at early stages (Figure 7C, left). Subcluster‐level analysis further showed that Arg1^+^, Cdk^+^, and Cxcr4^+^ macrophages were mainly located at late pseudotime stages, whereas Il1b^+^ and Irf8^+^ macrophages were enriched at early stages (Figure 7C, right). Lipid metabolism scores increased along pseudotime, suggesting that lipid metabolic reprogramming is closely linked to macrophage differentiation trajectory (Figure 7D).

We next quantified lipid metabolism, senescence, and efferocytosis scores across macrophage subclusters. Pro‐inflammatory Il1b+ macrophages showed lower lipid metabolism and efferocytosis scores than anti‐inflammatory Arg1^+^ macrophages, whereas their senescence scores were higher (Figure 7E–G). Stratifying macrophages by lipid metabolism score identified 459 downregulated and 470 upregulated genes between the low‐ and high‐lipid‐metabolism groups (Figure S4C). KEGG enrichment highlighted lysosomal function, endocytosis, and cellular senescence (Figure S4D), further linking lipid metabolic state to macrophage clearance function and senescence.

We further examined the expression of lipid metabolism‐related hub genes across macrophage subclusters. PPT1 was predominantly expressed in M1‐like macrophages, whereas TIAM2 was enriched in M2‐like macrophages; the other hub genes showed minimal expression in this dataset (Figure S5A). Key lipid homeostasis genes were markedly lower in pro‐inflammatory macrophages, including Il1b^+^, Il6^+^, and Irf8^+^ subclusters than in other macrophage subsets (Figure S5B). These results reinforce the tight relationship between macrophage lipid homeostasis, senescence, efferocytosis and inflammatory phenotype.

To test whether senescence mediates the effect of lipid dysregulation on macrophage efferocytosis, we performed rescue experiments in the context of LPS environment (20 ng/mL) using the p53 inhibitor PFT‐β (20 μM). Palmitic acid (PA) (200 μM) treatment increased lipid accumulation, lipid peroxidation, senescent‐cell proportion, and inflammatory markers, while reducing the efferocytosis index (Figure 7H,I). PFT‐β markedly attenuated these changes, supporting an important role of senescence‐associated signalling in PA‐induced efferocytosis impairment and inflammatory activation. Consistently, PA upregulated the mRNA expression of pro‐inflammatory genes, including *iNos, Il1b*, and *Tnfα*, and the senescence marker *Cdkn1a*, while downregulating the anti‐inflammatory gene *Il10* and the efferocytosis‐related gene *Mertk*. These transcriptional alterations were partially reversed by PFT‐β (Figure 7J).

Together, these data indicate that lipid accumulation drives macrophage inflammatory activation and efferocytosis impairment through a senescence‐dependent mechanism.

### 3.8 Rosuvastatin Restores Macrophage Lipophagy, Attenuates Senescence, and Rescues Efferocytosis

**FIGURE 8:**
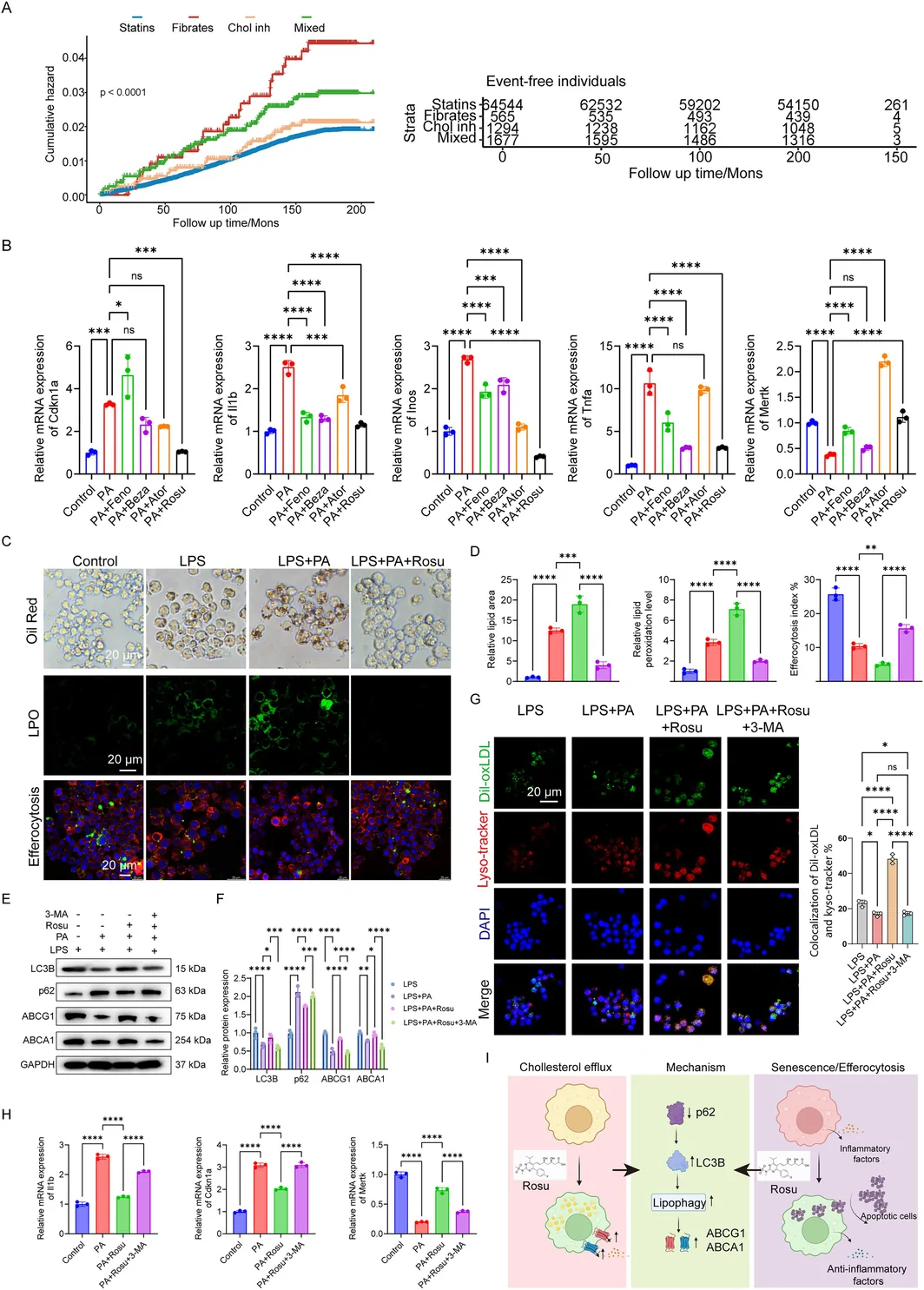
Statins improve cellular lipid homeostasis by regulating lipid autophagy, alleviate macrophage senescence, and attenuate inflammatory phenotype and phagocytic ability. (A) Survival analysis curve of the association between lipid‐lowering drug use and IVDD incidence risk based on the UKB database; (B) QPCR bar chart of lipid‐accumulated BMDM cells treated with statins, fibrates, and cholesterol inhibitors, respectively (*n* = 3); (C) oil red staining, lipid peroxidation staining, and efferocytosis staining of BMDM cells in each group; (D) quantitative analysis of staining images (*n* = 3); (E and F) WB images and quantitation analysis of autophagy markers LC3B and p62, and lipid homeostasis markers ABCG1 and ABCA1 (*n* = 3); (G) fluorescence images of lipid droplets and lysosomes in macrophages (*n* = 3); (H) PCR bar chart of the expression of inflammation, senescence and efferocytosis‐related genes in BMDM cells of each group (*n* = 3); (I) schematic diagram showing that statins improve cellular lipid homeostasis by regulating lipophagy, alleviate macrophage senescence, and improve inflammatory phenotype and efferocytosis capacity. *p < 0.05, **p < 0.01, ***p < 0.001, ****p < 0.0001.

These findings suggested that the lipid metabolism‐macrophage axis could be therapeutically targeted in IVDD. We first examined IVDD risk among UKB participants receiving different lipid‐lowering drug classes. Compared with other lipid‐lowering strategies, statin use was associated with a lower risk of IVDD (Figure 8A). Subsequently, we further verified at the cellular level by treating lipid‐accumulated BMDM macrophages with statins, fibrates, and cholesterol inhibitors, respectively (all 10 ng/mL), to explore macrophage senescence, efferocytosis and inflammatory phenotypes [38]. Among these interventions, rosuvastatin produced the strongest transcriptional correction of lipid homeostasis and macrophage dysfunction (Figure 8B) and was therefore selected for subsequent mechanistic experiments. Oil Red O staining, lipid peroxide staining, and efferocytosis staining showed that lipid metabolism disorder exacerbated lipid accumulation in macrophages under LPS stimulation (10 ng/mL), increased intracellular lipid peroxide levels, and reduced the efferocytosis capacity of macrophages (Figure 8C,D). These results suggest that lipid overload promotes a dysfunctional senescent and pro‐inflammatory macrophage phenotype. However, these detrimental effects were alleviated by rosuvastatin treatment (Figure 8C,D).

Given the central role of cholesterol flux and lysosomal degradation in macrophage lipid homeostasis, we next assessed whether rosuvastatin restored lipophagy. PA treatment decreased LC3B and increased p62, indicating impaired autophagic activity under lipid metabolic stress. Rosuvastatin restored LC3B and p62 expression patterns (Figure 8E,F). PA also reduced the cholesterol efflux proteins ABCA1 and ABCG1, whereas rosuvastatin rescued their expression. Notably, the protective effects of rosuvastatin were substantially blocked by 3‐methyladenine (3‐MA), an early‐stage autophagy inhibitor. Fluorescence imaging further showed that rosuvastatin enhanced lipid droplet‐lysosome co‐localization and reduced lipid droplet accumulation in activated macrophages, effects that were reversed by 3‐MA pretreatment (Figure 8G,H). Gene expression analysis confirmed that rosuvastatin attenuated lipotoxicity‐induced senescence, inflammation, and efferocytosis impairment, whereas 3‐MA abolished these benefits (Figure 8H).

Together, these results suggest that rosuvastatin restores cholesterol flux and lipophagic activity, thereby alleviating macrophage senescence, suppressing inflammatory activation and rescuing efferocytosis (Figure 8I).

### 3.9 Local Rosuvastatin Delivery Remodels the Immune‐Inflammatory Microenvironment and Alleviates IVDD In Vivo

**FIGURE 9:**
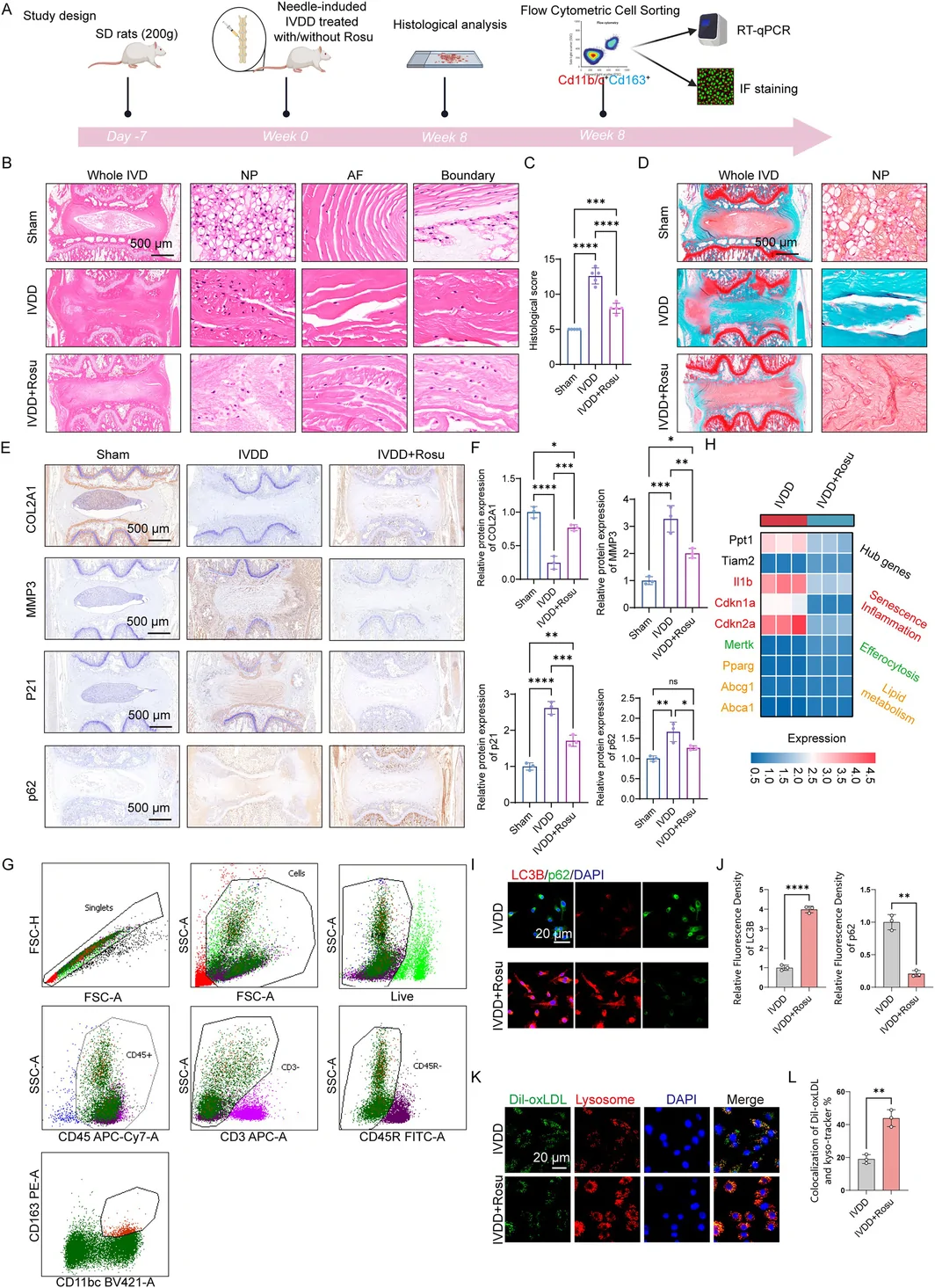
Lipid homeostasis‐regulating drugs can improve the local immune aging microenvironment and alleviate the progression of IVDD in vivo. (A) Schematic diagram of the animal experiment; (B) HE staining of rat caudal vertebrae; (C) quantitative analysis of HE staining of rat caudal vertebrae (*n* = 5); (D) SOFG staining of rat caudal vertebrae; (E) immunohistochemical staining of rat caudal vertebrae; (F) quantitative analysis of immunohistochemical staining of rat caudal vertebrae (*n* = 3); (G) schematic diagram of flow sorting of macrophages from rat intervertebral disc tissues; (H) PCR result heatmap of sorted macrophages from rat intervertebral discs; (I and J) fluorescence images of autophagy indicators in sorted macrophages from rat intervertebral discs of each group (*n* = 3); (K and L) co‐fluorescence images of lipid droplets and lysosomes in sorted macrophages from rat intervertebral discs of each group (*n* = 3). *p < 0.05, **p < 0.01, ***p < 0.001, ****p < 0.0001.

To verify whether restoration of lipid homeostasis alleviates IVDD in vivo, we established a rat caudal disc puncture model and administered rosuvastatin locally (Figure 9A). Haematoxylin and eosin (H&E) staining at the experimental endpoint showed severe structural disruption in the IVDD group, including loss of NP tissue and disorganisation of AF architecture (Figure 9B,C). In contrast, rosuvastatin treatment preserved disc structure, with improved NP retention, more ordered AF fibres, and better integrity of the junctional region.

SOFG staining showed marked loss of proteoglycans in the IVDD group, whereas rosuvastatin treatment restored proteoglycan‐rich areas (Figure 9D). Immunohistochemistry further showed that COL2A1 expression was significantly reduced in IVDD discs, whereas MMP3, p21, and PPT1 were elevated compared with Sham controls (all *p* < 0.001). Rosuvastatin increased COL2A1 expression and reduced MMP3, p21, and PPT1 levels relative to the IVDD group (*p* < 0.05 to *p* < 0.001) (Figure 9E,F). These results indicate that rosuvastatin mitigates ECM imbalance, cellular senescence, and abnormal hub‐gene expression during IVDD progression.

To further determine whether rosuvastatin modulated macrophage senescence in vivo, we isolated disc‐derived macrophages from rat intervertebral disc tissues by ex vivo flow cytometric sorting. After exclusion of debris, doublets, dead cells, CD3^+^ T cells, and CD45R^+^ B cells, macrophage‐enriched cells were sorted as live CD45^+^CD3^−^CD45R^−^CD11b/c^+^CD163^+^ cells for qRT‐PCR and ex vivo cytological analyses (Figures 9G and S6A). The results of macrophage gene expression showed that the expressions of macrophage senescence and inflammation‐related genes (*Cdkn1a, Cdkn2a, Il1b*) in the treatment group were significantly lower than those in the model group. In contrast, the expression levels of lipid metabolism‐related genes, including *Pparg, Abca1*, and *Abcg1*, and the efferocytosis‐related gene *Mertk* were increased after rosuvastatin treatment (Figure 9H). We also evaluated the expression of hub genes, and the results showed that rosuvastatin treatment similarly ameliorated the expression changes of *Ppt1* and *Tiam2* (Figure 9H). Meanwhile, we collected macrophages from each group for in vitro culture and staining. Cellular polarisation staining indicated that after rosuvastatin treatment, the expression of the anti‐inflammatory gene Arg1 in macrophages was increased, while the expression of the pro‐inflammatory gene iNOS was decreased (Figure S6B,C). These findings provide cell‐type‐specific evidence that rosuvastatin alleviates macrophage senescence and functional impairment in the degenerative disc microenvironment.

We further evaluated macrophage lipophagy in vivo. Compared with the IVDD group, rosuvastatin increased LC3B and decreased p62 expression (Figure 9I,J), indicating improved autophagic activity. Lipid droplet‐lysosome co‐localization analysis showed enhanced lysosomal clearance of lipid droplets after rosuvastatin treatment (Figure 9K,L), suggesting that rosuvastatin remodels macrophage function by activating lipophagy‐mediated lipid degradation.

Together, these in vivo data indicate that local rosuvastatin delivery improves macrophage lipid metabolism, senescence, inflammation, and efferocytosis, thereby remodelling the disc immune microenvironment and slowing IVDD progression.

**FIGURE 10:**
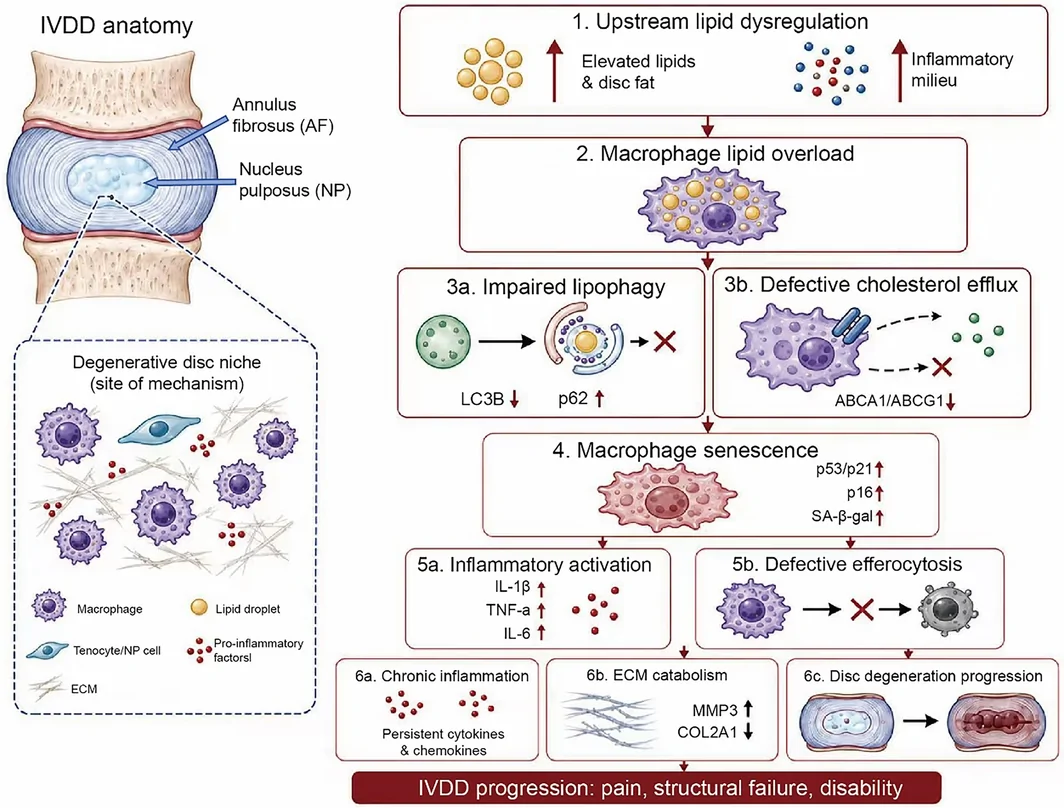
Schematic illustration of the mechanism by which macrophage lipid overload drives IVDD.

Overall, these results support a mechanistic model in which lipid dysregulation within the degenerative disc niche induces macrophage lipid overload, thereby impairing lipophagy and cholesterol efflux and promoting macrophage senescence (Figure 10). Senescent lipid‐laden macrophages may amplify inflammatory responses and defective efferocytosis, leading to persistent inflammation, extracellular matrix degradation, and progressive disc degeneration. This model provides a conceptual framework linking macrophage lipid metabolic dysfunction to IVDD progression.

## 4 Discussion

Our multidimensional study supports a macrophage‐centred pathogenic circuit in IVDD: the Lipotoxicity‐Senescence‐Inflammation (LSI) axis. Our data suggest that macrophage lipid homeostasis is closely linked to IVDD progression and may actively contribute to disease‐associated immune dysfunction, in which lipotoxicity promotes cellular senescence, impaired efferocytosis and inflammatory activation. This LSI axis establishes a coherent framework that explains how metabolic imbalance actively orchestrates immune failure to perpetuate disc degeneration. Crucially, we identify statin‐mediated restoration of lipophagic flux as a therapeutically viable strategy to break this vicious cycle, thereby shifting the paradigm from symptomatic management to targeted immune‐metabolic intervention.

From the perspective of cell behaviour regulation, our study demonstrates that lipid metabolic dysregulation actively reshapes macrophage behaviour during IVDD progression. Rather than functioning as passive inflammatory responders, lipid‐overloaded macrophages acquire a pathological behavioural program characterised by impaired lipophagy, cellular senescence, SASP‐associated inflammatory activation and defective efferocytosis. This shift compromises their normal tissue‐clearing and inflammation‐resolving functions and converts macrophages into active drivers of the degenerative disc microenvironment. Importantly, our data further suggest that macrophage senescence represents a reversible cellular state rather than an irreversible endpoint of degeneration. In our analysis, Cluster 4 macrophages were enriched in IVDD tissues and were characterised by reduced lipid metabolic activity, increased senescence scores, and a more pro‐inflammatory polarisation state. This phenotype resembles senescent or dysfunctional macrophage populations reported in other chronic diseases. For example, senescent synovial macrophages in osteoarthritis have been associated with enhanced M1‐like inflammatory activation, mitochondrial dysfunction and impaired efferocytosis [39, 40]. Similarly, in atherosclerosis, lipid‐laden foam‐cell‐like macrophages can acquire senescence‐associated features, sustain chronic inflammatory signalling and exhibit defective clearance capacity [41, 42]. These similarities suggest that the Cluster 4 macrophages identified in IVDD may share a core dysfunctional program with macrophages described in other chronic degenerative diseases. However, because the intervertebral disc is exposed to distinct local cues, such as hypoxia or nutrient limitation, Cluster 4 found here may function as an IVDD‐associated macrophage phenotype rather than a direct counterpart of macrophage subsets in osteoarthritis or atherosclerosis. Further, pharmacological restoration of lipid homeostasis by rosuvastatin attenuated macrophage senescence, restored lipophagic activity, improved efferocytosis and reduced inflammatory activation. These findings indicate that intervention targeting macrophage senescence can reprogram detrimental macrophage behaviour toward a more homeostatic and reparative state. Thus, the present study provides a cell behaviour regulation‐based framework for IVDD therapy and highlights the lipid metabolism‐senescence axis as an actionable target for immune‐metabolic intervention.

Under physiological conditions, infiltrating macrophages can clear apoptotic cells via efferocytosis, thereby maintaining local tissue homeostasis [43]. Recent evidence has further highlighted macrophage efferocytosis as a potential therapeutic strategy for IVDD. Efficient clearance of apoptotic nucleus pulposus cells by macrophages prevents the secondary release of intracellular damage‐associated molecules, promotes inflammation resolution, and contributes to the restoration of disc tissue homeostasis [44]. Our findings extend this concept by showing that, in IVDD, this efferocytosis function is actively disrupted by a lipid‐dysregulated microenvironment, and infiltrating macrophages may undergo abnormal intracellular lipid accumulation, which further activates cellular senescence and chronic inflammation [45, 46]. We found that increased disc fat fraction and elevated plasma lipid levels in patients with advanced IVDD were associated with a microenvironment favouring macrophage lipid overload. Consistently, single‐cell RNA sequencing analysis showed that macrophages with low lipid metabolism scores exhibited higher senescence scores, a more M1‐like inflammatory phenotype, and reduced efferocytosis‐related signatures. These findings support a close relationship among lipid metabolic disturbance, macrophage senescence, and impaired efferocytosis during IVDD progression. The partial rescue of PA‐induced efferocytosis impairment by the p53 inhibitor PFT‐β further supports senescence as an important intermediary mechanism. Furthermore, intervention experiments with rosuvastatin supported the therapeutic feasibility of targeting this lipid metabolism–macrophage dysfunction axis. Rosuvastatin restored lipophagic activity, reduced toxic lipid accumulation, attenuated senescence‐related signalling, improved cholesterol efflux and partially rescued efferocytosis. These findings suggest that restoring macrophage lipid homeostasis may interrupt both senescence‐dependent and potentially senescence‐independent mechanisms of efferocytosis impairment. Thus, the proposed Lipotoxicity‐Senescence‐Inflammation axis should be viewed as a mechanistic framework in which macrophage senescence represents a major, experimentally supported, but not exclusive, mediator of lipid overload‐induced immune dysfunction in IVDD. These results align with prior studies showing that lipid disorders can induce macrophage senescence and inflammation [47]. Yang et al. also reported that macrophages could regulate hepatic homeostasis by promoting the autophagic degradation of lipid droplets [48, 49, 50]. Nevertheless, the current data do not fully exclude the possibility that lipid accumulation may also impair efferocytosis through senescence‐independent mechanisms. Excessive lipid loading may directly alter plasma membrane lipid composition and fluidity, interfere with cytoskeletal rearrangement required for engulfment, impair phagocytic receptor signalling, aggravate lysosomal dysfunction, and reduce cholesterol efflux, all of which may compromise the recognition, internalisation, and degradation of apoptotic cells. Therefore, although our data support a senescence‐dependent contribution to lipid‐induced efferocytosis impairment, macrophage senescence should not be interpreted as the sole mechanism responsible for defective efferocytosis under lipid overload conditions.

We also identified four lipid metabolism‐related hub genes associated with IVDD progression: TIAM2, SLC44A4, PPT1 and PTGDS. In our integrated transcriptomic and machine‐learning analyses, TIAM2, SLC44A4, and PPT1 were negatively associated with IVDD risk, whereas PTGDS was positively associated. These findings suggest that these genes may serve as candidate lipid metabolism‐related biomarkers reflecting disease‐associated immune‐metabolic remodelling in IVDD. TIAM2, a guanine nucleotide exchange factor that activates Rho GTPases, regulates cytoskeletal dynamics and cell migration [51]. Prior studies have linked TIAM2 to macrophage M2‐like polarisation and EphB‐dependent endocytic regulation [52]. Therefore, the reduced expression of TIAM2 observed in IVDD tissues may be associated with impaired macrophage homeostatic or clearance‐related phenotypes. Nevertheless, whether TIAM2 directly regulates macrophage efferocytosis, senescence, or inflammatory polarisation in the degenerative disc microenvironment remains unknown and requires experimental validation. SLC44A4 is a solute carrier family member involved in choline transport, and related solute transport pathways have been implicated in lipid uptake, endoplasmic reticulum stress, lipid droplet accumulation and cellular senescence [53, 54]. Based on these known biological associations, altered SLC44A4 expression may reflect changes in membrane lipid metabolism or lipid‐stress responses during IVDD progression. However, our current data do not establish a causal role for SLC44A4 in macrophage lipid handling, senescence induction, or efferocytosis impairment in IVDD. PPT1 encodes palmitoyl‐protein thioesterase 1, which is mainly localised in lysosomes and serves as a key enzyme catalysing the depalmitoylation of S‐palmitoylated proteins. PPT1 deficiency leads to an increase in lysosomal pH, thereby inducing lysosomal dysfunction [55]. In addition, PPT1 deficiency also leads to abnormal cellular lipid metabolism, causing massive lipid accumulation [56]. Weng et al. [57] revealed that macrophages with high PPT1 expression significantly upregulate the expression of immunosuppressive molecules (Galectin‐9, CD172a, IL‐10 and CCL28) and the M2 macrophage‐specific marker CCR2, suggesting that PPT1 may promote the transformation of macrophages toward an immunosuppressive phenotype. In our study, PPT1 expression was altered in IVDD and showed associations with macrophage‐related immune signatures. These observations support a possible link between PPT1, lysosomal lipid processing and macrophage functional remodelling. However, whether PPT1 directly controls lipophagy, macrophage senescence, or efferocytosis in IVDD has not yet been determined. PTGDS encodes lipocalin‐type prostaglandin D2 synthase, whose core function is to catalyse the conversion of prostaglandin H2 (PGH2) to prostaglandin D2, while promoting the polarisation of macrophages toward the pro‐inflammatory M1 phenotype. Zhong et al. [58] confirmed that PTGDS overexpression can inhibit the activation of the PI3K/AKT signalling pathway, thereby blocking the polarisation of macrophages toward the M2 phenotype. Consistent with this background, the positive association between PTGDS expression and IVDD risk in our analysis suggests that PTGDS may mark an inflammatory lipid mediator‐related state in degenerative discs. However, direct evidence demonstrating that PTGDS drives macrophage senescence, defective efferocytosis, or IVDD progression is still lacking. Therefore, TIAM2, SLC44A4, PPT1, and PTGDS may represent lipid metabolism‐related hub genes associated with macrophage immune‐metabolic remodelling during IVDD progression. Rather than proving that these genes function as direct mechanistic regulators of the Lipotoxicity‐Senescence‐Inflammation axis, our data support their value as candidate biomarkers and potential entry points for future mechanistic studies. Further gain‐ and loss‐of‐function experiments in macrophages, together with in vivo validation in IVDD models, will be required to determine whether these genes causally regulate macrophage senescence, efferocytosis, lipid homeostasis and disc degeneration.

Based on the lipid homeostasis and macrophage function, we investigated the therapeutic vulnerability using statins, and found that population data from the UK Biobank indicated a lower risk of IVDD among statin users. In addition, we demonstrated that rosuvastatin showed the best effects. Mechanistically, rosuvastatin did not simply act as a lipid‐lowering agent; it restored macrophage cholesterol efflux, enhanced lipophagic flux, rescued efferocytosis, and promoted a less inflammatory macrophage phenotype. The autophagy inhibitor 3‐MA attenuated these effects, supporting lipophagy as a key mediator. In vivo, local rosuvastatin delivery improved histopathology, restored proteoglycan content, rebalanced ECM metabolism, and reduced senescence and inflammatory markers in disc‐derived macrophages. Beyond IVDD, the LSI axis may represent a broader pathological framework in age‐related degenerative diseases characterised by lipid dysregulation, cellular senescence, and chronic inflammation, such as atherosclerosis and osteoarthritis [59, 60]. This shifts the therapeutic focus from replacing lost cells to reprogramming the dysfunctional immune‐metabolic niche. Nevertheless, it should be acknowledged that the protective effects of rosuvastatin should not be attributed exclusively to the restoration of macrophage lipid homeostasis. Statins are known to exert pleiotropic biological activities beyond lipid regulation, including anti‐inflammatory, immunomodulatory, antioxidant and cytoprotective effects. Therefore, rosuvastatin may also alleviate IVDD by suppressing inflammatory signalling, and future studies using macrophage‐specific lipid metabolism interventions will be required.

Lipophagy and cellular senescence are closely linked under lipotoxic stress [61]. Defective lipophagy may cause lipid droplet accumulation, impaired cholesterol efflux, lysosomal dysfunction, and lipid peroxidation, thereby activating senescence‐related pathways such as the p53/p21 axis and promoting SASP‐associated inflammatory signalling [62]. Conversely, senescent macrophages may further exhibit impaired lysosomal and autophagic activity, which aggravates lipid retention and sustains inflammatory activation [63]. Therefore, impaired lipophagy and macrophage senescence may form a self‐amplifying pathological loop that contributes to defective efferocytosis and chronic inflammation in IVDD [44]. Restoration of lipophagy may therefore serve as a promising therapeutic strategy. By reducing lipid overload, improving cholesterol homeostasis, alleviating macrophage senescence, and restoring efferocytosis, lipophagy‐targeted intervention may help resolve inflammation and slow IVDD progression. Our findings that rosuvastatin restored lipophagic activity and attenuated macrophage senescence further support the therapeutic potential of targeting the lipophagy‐senescence axis in IVDD [64]. Notably, senescent macrophages and their SASP‐associated factors could be explored as candidate biomarkers for early IVDD, although further validation in larger clinical cohorts and more accessible sample types is required. Moreover, our findings that rosuvastatin restored lipid homeostasis, reduced senescence‐related signalling, rescued efferocytosis, and alleviated IVDD pathology suggest that macrophage senescence may also serve as a tractable therapeutic target for immune‐metabolic intervention.

Several limitations should be acknowledged. First, although TIAM2, SLC44A4, PPT1, and PTGDS were identified as lipid metabolism‐related hub genes associated with IVDD progression, their biological functions in regulating macrophage lipid homeostasis, senescence, and efferocytosis have not been directly validated in the present study. Therefore, the mechanistic roles of these genes should be interpreted with caution and require further confirmation using gene‐specific gain‐ and loss‐of‐function models in macrophages and in vivo IVDD models. Second, the crosstalk between senescent macrophages and other disc‐resident cells, especially NP cells, should be investigated to define how SASP factors shape the degenerative cellular ecosystem. Third, although flow‐sorted disc macrophage analysis provided cell‐type‐specific quantitative evidence, this study did not include in situ co‐staining of macrophage markers with senescence markers in rat disc tissues. Future studies using optimised immunofluorescence co‐localization or spatial transcriptomic approaches will be helpful to further validate the spatial relationship between macrophage senescence and rosuvastatin‐mediated protection in vivo. Finally, the initial LASSO model showed an exceptionally high AUC in the discovery dataset, which may reflect optimistic performance estimation. Although cross‐validation, multi‐algorithm screening, SHAP interpretation, calibration analysis, decision curve analysis, and clinical impact curve assessment were used to improve robustness, the predictive value of the four‐gene signature still requires validation in independent external cohorts with larger sample sizes and standardised clinical annotations.

## 5 Conclusion

In conclusion, this study defines macrophage lipid homeostasis as a central regulator of IVDD through a senescence‐dependent impairment of efferocytosis. These findings highlight lipid‐induced macrophage senescence as a reversible and druggable cellular state, providing a cell behaviour regulation‐based strategy for immune‐metabolic intervention in IVDD (Figure 10).

## Author Contributions

**Jinyu Wang**, **Shiyong Ling**, **Qi Wang** and **Xuege Qiao:** investigation and the original manuscript writing. **Jianpeng Xing**, **Siyang Ji** and **Linhui Han:** data collection. **Chen Yan** and **Zhishen Niu:** software. **Lei Yuan**, **Baolian Zhao**, **Kaiqiang Sun** and **Jiangang Shi:** design, funding and manuscript reviewing.

## Funding

This study was supported by the National Natural Science Foundation of China (Grant No. 82172381, 82302760, 82472493).

## Conflicts of Interest

The authors declare no conflicts of interest.

## Supporting information


**Figure S1:** (A) Venn diagram illustrating the common genes between DEGs and LMGs; (B) LASSO coefficient curve plot; (C) LASSO partial likelihood deviation plot; (D) ROC curve of the LASSO model; (E) risk scores of the control group and IVDD group; (F–H) summary plots of Catboost, LightGBM and XGboost; (I–K) Swarm plots of Catboost, LightGBM and XGboost.
**Figure S2:** (A) KEGG pathway enrichment plot of the TIAM2 gene; (B) KEGG pathway enrichment plot of the SLC44A4 gene; (C) KEGG pathway enrichment plot of the PTGDS gene; (D) KEGG pathway enrichment plot of the PPT1 gene.
**Figure S3:** (A) Bubble plot of marker genes of different intervertebral disc cell subgroups; (B) heatmap of marker gene expression in each macrophage subgroup.
**Figure S4:** (A) Proportion distribution of various macrophage subgroups; (B) expression profiles of marker genes of macrophage subgroups; (C) Volcano plot of differentially expressed genes grouped by lipid metabolism level; (D) KEGG enrichment pathway analysis of differentially expressed genes grouped by lipid metabolism level.
**Figure S5:** (A) Box plot of PPT1 and TIAM2 expression in macrophages with different phenotypes; (B) Bubble plot of key lipid metabolism genes expressed in different macrophage subgroups.
**Figure S6:** (A) Schematic diagram of flow sorting of rat intervertebral disc tissues; (B) polarisation fluorescence images of sorted macrophages from rat intervertebral discs of each group; (C) quantitative analysis of macrophage polarisation fluorescence images.
**Table S1:** The detailed information of imaging data.
**Table S2:** The detailed information of blood routine test results.
**Table S3:** The detailed information of intervertebral disc samples.

## Data Availability

The datasets described and analysed during the current study are available from the corresponding author upon reasonable request.

## References

[1] N. Fine , S. Lively , C. A. Séguin , A. V. Perruccio , M. Kapoor , and R. Rampersaud , “Intervertebral Disc Degeneration and Osteoarthritis: A Common Molecular Disease Spectrum,” Nature Reviews Rheumatology 19, no. 3 (2023): 136–152. 36702892 10.1038/s41584-022-00888-z

[2] J. Hartvigsen , M. J. Hancock , A. Kongsted , et al., “What Low Back Pain Is and Why We Need to Pay Attention,” Lancet 391, no. 10137 (2018): 2356–2367. 29573870 10.1016/S0140-6736(18)30480-X

[3] S. Chen , M. Chen , X. Wu , et al., “Global, Regional and National Burden of Low Back Pain 1990‐2019: A Systematic Analysis of the Global Burden of Disease Study 2019,” Journal of Orthopaedic Translation 32 (2022): 49–58. 34934626 10.1016/j.jot.2021.07.005 PMC8639804

[4] Y. Dou , Y. Zhang , Y. Liu , et al., “Role of Macrophage in Intervertebral Disc Degeneration,” Bone Research 13, no. 1 (2025): 15. 39848963 10.1038/s41413-024-00397-7 PMC11758090

[5] L. Kang , H. Zhang , C. Jia , R. Zhang , and C. Shen , “Epigenetic Modifications of Inflammation in Intervertebral Disc Degeneration,” Ageing Research Reviews 87 (2023): 101902. 36871778 10.1016/j.arr.2023.101902

[6] B. G. Ashinsky , S. E. Gullbrand , C. Wang , et al., “Degeneration Alters Structure‐Function Relationships at Multiple Length‐Scales and Across Interfaces in Human Intervertebral Discs,” Journal of Anatomy 238, no. 4 (2021): 986–998. 33205444 10.1111/joa.13349 PMC7930769

[7] G. Kaneda , L. Zila , J. T. Wechsler , et al., “What a Pain in the Back: Etiology, Diagnosis and Future Treatment Directions for Discogenic Low Back Pain,” Bone Research 13, no. 1 (2025): 89. 41120264 10.1038/s41413-025-00472-7 PMC12541100

[8] L. J. Smith , N. L. Nerurkar , K. S. Choi , B. D. Harfe , and D. M. Elliott , “Degeneration and Regeneration of the Intervertebral Disc: Lessons From Development,” Disease Models & Mechanisms 4, no. 1 (2011): 31–41. 21123625 10.1242/dmm.006403 PMC3008962

[9] M. V. Risbud and I. M. Shapiro , “Role of Cytokines in Intervertebral Disc Degeneration: Pain and Disc Content,” Nature Reviews Rheumatology 10, no. 1 (2014): 44–56. 24166242 10.1038/nrrheum.2013.160 PMC4151534

[10] K. G. Burt , M. Kim , D. C. Viola , A. C. Abraham , and N. O. Chahine , “Nuclear Factor κB Overactivation in the Intervertebral Disc Leads to Macrophage Recruitment and Severe Disc Degeneration,” Science Advances 10, no. 23 (2024): eadj3194. 38848366 10.1126/sciadv.adj3194 PMC11160472

[11] Q. Xiang , J. Zhan , S. Tian , et al., “Human iPSCs Derived MSCs‐Secreted Exosomes Modulate Senescent Nucleus Pulposus Cells Induced Macrophage Polarisation via Metabolic Reprogramming to Mitigate Intervertebral Disc Degeneration,” Advanced Science 12, no. 36 (2025): e04347. 40619582 10.1002/advs.202504347 PMC12463037

[12] J. Yan and T. Horng , “Lipid Metabolism in Regulation of Macrophage Functions,” Trends in Cell Biology 30, no. 12 (2020): 979–989. 33036870 10.1016/j.tcb.2020.09.006

[13] M. Li , Y. Yang , L. Xiong , P. Jiang , J. Wang , and C. Li , “Metabolism, Metabolites, and Macrophages in Cancer,” Journal of Hematology & Oncology 16, no. 1 (2023): 80. 37491279 10.1186/s13045-023-01478-6 PMC10367370

[14] P. S. Liu , Y. T. Chen , X. Li , et al., “CD40 Signal Rewires Fatty Acid and Glutamine Metabolism for Stimulating Macrophage Anti‐Tumorigenic Functions,” Nature Immunology 24, no. 3 (2023): 452–462. 36823405 10.1038/s41590-023-01430-3 PMC9977680

[15] N. Shao , H. Qiu , J. Liu , et al., “Targeting Lipid Metabolism of Macrophages: A New Strategy for Tumor Therapy,” Journal of Advanced Research 68 (2025): 99–114. 38373649 10.1016/j.jare.2024.02.009 PMC11785569

[16] R. Liu , Y. Chen , W. Fu , et al., “Fexofenadine Inhibits TNF Signaling Through Targeting to Cytosolic Phospholipase A2 and Is Therapeutic Against Inflammatory Arthritis,” Annals of the Rheumatic Diseases 78, no. 11 (2019): 1524–1535. 31302596 10.1136/annrheumdis-2019-215543 PMC8157820

[17] M. Cuchel and D. J. Rader , “Macrophage Reverse Cholesterol Transport: Key to the Regression of Atherosclerosis,” Circulation 113, no. 21 (2006): 2548–2555. 16735689 10.1161/CIRCULATIONAHA.104.475715

[18] J. Yi , Q. Zhou , J. Huang , S. Niu , G. Ji , and T. Zheng , “Lipid Metabolism Disorder Promotes the Development of Intervertebral Disc Degeneration,” Biomedicine & Pharmacotherapy 166 (2023): 115401. 37651799 10.1016/j.biopha.2023.115401

[19] M. Rosas‐Ballina , X. L. Guan , A. Schmidt , and D. Bumann , “Classical Activation of Macrophages Leads to Lipid Droplet Formation Without De Novo Fatty Acid Synthesis,” Frontiers in Immunology 11 (2020): 131. 32132994 10.3389/fimmu.2020.00131 PMC7040478

[20] J. Tan , M. Shi , B. Li , Y. Liu , S. Luo , and X. Cheng , “Role of Arachidonic Acid Metabolism in Intervertebral Disc Degeneration: Identification of Potential Biomarkers and Therapeutic Targets via Multi‐Omics Analysis and Artificial Intelligence Strategies,” Lipids in Health and Disease 22, no. 1 (2023): 204. 38007425 10.1186/s12944-023-01962-5 PMC10675942

[21] K. J. Moore , F. J. Sheedy , and E. A. Fisher , “Macrophages in Atherosclerosis: A Dynamic Balance,” Nature Reviews. Immunology 13, no. 10 (2013): 709–721. 10.1038/nri3520 PMC4357520 23995626

[22] X. W. Liu , H. W. Xu , Y. Y. Yi , S. B. Zhang , and S. J. Wang , “Role of Ferroptosis and Immune Infiltration in Intervertebral Disc Degeneration: Novel Insights From Bioinformatics Analyses,” Frontiers in Cell and Developmental Biology 11 (2023): 1170758. 37736497 10.3389/fcell.2023.1170758 PMC10509768

[23] C. Laliberté , B. Bossé , V. Bourdeau , et al., “Senescent Macrophages Release Inflammatory Cytokines and RNA‐Loaded Extracellular Vesicles to Circumvent Fibroblast Senescence,” Biomedicine 12, no. 5 (2024): 1089. 10.3390/biomedicines12051089 PMC11118806 38791051

[24] Z. Salloum , K. Dauner , Y. F. Li , et al., “Statin‐Mediated Reduction in Mitochondrial Cholesterol Primes an Anti‐Inflammatory Response in Macrophages by Upregulating Jmjd3,” eLife 13 (2024): 13. 10.7554/eLife.85964 PMC11186637 38602170

[25] C. Y. Liu , C. A. McKenzie , H. Yu , J. H. Brittain , and S. B. Reeder , “Fat Quantification With IDEAL Gradient Echo Imaging: Correction of Bias From T(1) and Noise,” Magnetic Resonance in Medicine 58, no. 2 (2007): 354–364. 17654578 10.1002/mrm.21301

[26] S. Islam , U. Kjällquist , A. Moliner , et al., “Characterization of the Single‐Cell Transcriptional Landscape by Highly Multiplex RNA‐Seq,” Genome Research 21, no. 7 (2011): 1160–1167. 21543516 10.1101/gr.110882.110 PMC3129258

[27] J. H. Zhao , D. Stacey , N. Eriksson , et al., “Genetics of Circulating Inflammatory Proteins Identifies Drivers of Immune‐Mediated Disease Risk and Therapeutic Targets,” Nature Immunology 24, no. 9 (2023): 1540–1551. 37563310 10.1038/s41590-023-01588-w PMC10457199

[28] L. Ottensmann , R. Tabassum , S. E. Ruotsalainen , et al., “Genome‐Wide Association Analysis of Plasma Lipidome Identifies 495 Genetic Associations,” Nature Communications 14, no. 1 (2023): 6934. 10.1038/s41467-023-42532-8 PMC10618167 37907536

[29] N. M. Davies , M. V. Holmes , and G. Davey Smith , “Reading Mendelian Randomisation Studies: A Guide, Glossary, and Checklist for Clinicians,” BMJ 362 (2018): k601. 30002074 10.1136/bmj.k601 PMC6041728

[30] X. Zhao , J. Zhang , J. Liu , J. Yuan , T. Wu , and X. Cheng , “Comprehensive Analysis of Gene Expression Profiles of Annulus Fibrosus Subtypes and Hub Genes in Intervertebral Disc Degeneration,” Aging 16, no. 6 (2024): 5370–5386. 38484139 10.18632/aging.205653 PMC11006460

[31] S. Chen , X. Yang , H. Gu , et al., “Predictive Etiological Classification of Acute Ischemic Stroke Through Interpretable Machine Learning Algorithms: A Multicenter, Prospective Cohort Study,” BMC Medical Research Methodology 24, no. 1 (2024): 199. 39256656 10.1186/s12874-024-02331-1 PMC11384709

[32] A. Subramanian , P. Tamayo , V. K. Mootha , et al., “Gene Set Enrichment Analysis: A Knowledge‐Based Approach for Interpreting Genome‐Wide Expression Profiles,” Proceedings of The National Academy of Sciences of The United States of America 102, no. 43 (2005): 15545–15550. 16199517 10.1073/pnas.0506580102 PMC1239896

[33] G. Toda , T. Yamauchi , T. Kadowaki , and K. Ueki , “Preparation and Culture of Bone Marrow‐Derived Macrophages From Mice for Functional Analysis,” STAR Protocols 2, no. 1 (2021): 100246. 33458708 10.1016/j.xpro.2020.100246 PMC7797923

[34] K. Sun , C. Yan , X. Dai , et al., “Catalytic Nanodots‐Driven Pyroptosis Suppression in Nucleus Pulposus for Antioxidant Intervention of Intervertebral Disc Degeneration,” Advanced Materials 36, no. 19 (2024): e2313248. 38299823 10.1002/adma.202313248

[35] Q. Zhang , Z. Li , S. Zhou , and J. Li , “Regenerative Outcomes of Combining siCOL1A2 Hydrogel With Acupuncture in a Rat Model of Chronic Intervertebral Disc Degeneration,” Bioengineering 11, no. 11 (2024): 1066. 39593726 10.3390/bioengineering11111066 PMC11591507

[36] I. A. Salladay‐Perez , I. Avila , L. Estrada , et al., “p21+TREM2+ Senescent Macrophages Fuel Inflammaging and Metabolic Dysfunction‐Associated Steatotic Liver Disease,” Nature Aging 6, no. 4 (2026): 792–815. 41991686 10.1038/s43587-026-01101-6 PMC13099426

[37] W. Wang , R. L. Xu , P. He , and R. Chen , “MAR1 Suppresses Inflammatory Response in LPS‐Induced RAW 264.7 Macrophages and Human Primary Peripheral Blood Mononuclear Cells via the SIRT1/PGC‐1α/PPAR‐γ Pathway,” Journal of Inflammation 18, no. 1 (2021): 8. 33557833 10.1186/s12950-021-00271-x PMC7869219

[38] C. Furman , C. Copin , M. Kandoussi , et al., “Rosuvastatin Reduces MMP‐7 Secretion by Human Monocyte‐Derived Macrophages: Potential Relevance to Atherosclerotic Plaque Stability,” Atherosclerosis 174, no. 1 (2004): 93–98. 15135256 10.1016/j.atherosclerosis.2004.01.009

[39] Y. Liu , J. Duan , Y. Dang , et al., “Remodeling of Senescent Macrophages in Synovium Alleviates Trauma‐ and Aging‐Induced Osteoarthritis,” Bioactive Materials 55 (2026): 42–56. 41035432 10.1016/j.bioactmat.2025.09.016 PMC12481617

[40] L. Del Sordo , G. B. Blackler , H. T. Philpott , et al., “Impaired Efferocytosis by Synovial Macrophages in Patients With Knee Osteoarthritis,” Arthritis and Rheumatology 75, no. 5 (2023): 685–696. 36448607 10.1002/art.42412

[41] B. G. Childs , D. J. Baker , T. Wijshake , C. A. Conover , J. Campisi , and J. M. van Deursen , “Senescent Intimal Foam Cells Are Deleterious at All Stages of Atherosclerosis,” Science 354, no. 6311 (2016): 472–477. 27789842 10.1126/science.aaf6659 PMC5112585

[42] U. K. Dhawan , T. Vartak , H. Englert , et al., “Macrophage DNases Limit Neutrophil Extracellular Trap‐Mediated Defective Efferocytosis in Atherosclerosis,” Circulation Research 137, no. 10 (2025): 1255–1275. 41031413 10.1161/CIRCRESAHA.125.326353 PMC12542999

[43] B. D. Gerlach , P. B. Ampomah , A. Yurdagul , et al., “Efferocytosis Induces Macrophage Proliferation to Help Resolve Tissue Injury,” Cell Metabolism 33, no. 12 (2021): 2445–2463. 34784501 10.1016/j.cmet.2021.10.015 PMC8665147

[44] S. Chen , H. Zhang , Z. Wang , et al., “Macrophage Efferocytosis as a Therapeutic Strategy in Intervertebral Disc Degeneration,” Cell Proliferation 58, no. 10 (2025): e70068. 40488295 10.1111/cpr.70068 PMC12508695

[45] B. Li , Z. Xin , S. Gao , et al., “SIRT6‐Regulated Macrophage Efferocytosis Epigenetically Controls Inflammation Resolution of Diabetic Periodontitis,” Theranostics 13, no. 1 (2023): 231–249. 36593966 10.7150/thno.78878 PMC9800730

[46] Y. Wang , J. Wang , J. Zhang , et al., “Stiffness Sensing via Piezo1 Enhances Macrophage Efferocytosis and Promotes the Resolution of Liver Fibrosis,” Science Advances 10, no. 23 (2024): eadj3289. 38838160 10.1126/sciadv.adj3289 PMC11152137

[47] A. Yurdagul , M. Subramanian , X. Wang , et al., “Macrophage Metabolism of Apoptotic Cell‐Derived Arginine Promotes Continual Efferocytosis and Resolution of Injury,” Cell Metabolism 31, no. 3 (2020): 518–533. 32004476 10.1016/j.cmet.2020.01.001 PMC7173557

[48] R. Terao , T. J. Lee , J. Colasanti , et al., “LXR/CD38 Activation Drives Cholesterol‐Induced Macrophage Senescence and Neurodegeneration via NAD+ Depletion,” Cell Reports 43, no. 5 (2024): 114102. 38636518 10.1016/j.celrep.2024.114102 PMC11223747

[49] T. Yang , X. Qu , X. Wang , et al., “The Macrophage STING‐YAP Axis Controls Hepatic Steatosis by Promoting the Autophagic Degradation of Lipid Droplets,” Hepatology 80, no. 5 (2024): 1169–1183. 37870294 10.1097/HEP.0000000000000638 PMC11035483

[50] A. R. Tall and L. Yvan‐Charvet , “Cholesterol, Inflammation and Innate Immunity,” Nature Reviews. Immunology 15, no. 2 (2015): 104–116. 10.1038/nri3793 PMC4669071 25614320

[51] J. Gong , T. N. Gaitanos , O. Luu , et al., “Gulp1 Controls Eph/Ephrin Trogocytosis and Is Important for Cell Rearrangements During Development,” Journal of Cell Biology 218, no. 10 (2019): 3455–3471. 31409653 10.1083/jcb.201901032 PMC6781437

[52] T. N. Gaitanos , J. Koerner , and R. Klein , “Tiam‐Rac Signaling Mediates Trans‐Endocytosis of Ephrin Receptor EphB2 and Is Important for Cell Repulsion,” Journal of Cell Biology 214, no. 6 (2016): 735–752. 27597758 10.1083/jcb.201512010 PMC5021091

[53] X. Chen , K. Chen , J. Hu , et al., “Palmitic Acid Induces Lipid Droplet Accumulation and Senescence in Nucleus Pulposus Cells via ER‐Stress Pathway,” Communications Biology 7, no. 1 (2024): 539. 38714886 10.1038/s42003-024-06248-9 PMC11076507

[54] Z. Ma , W. Xia , F. Liu , et al., “SLC44A4 Mutation Causes Autosomal Dominant Hereditary Postlingual Non‐Syndromic Mid‐Frequency Hearing Loss,” Human Molecular Genetics 26, no. 2 (2017): 383–394. 28013291 10.1093/hmg/ddw394

[55] E. Hellsten , J. Vesa , V. M. Olkkonen , A. Jalanko , and L. Peltonen , “Human Palmitoyl Protein Thioesterase: Evidence for Lysosomal Targeting of the Enzyme and Disturbed Cellular Routing in Infantile Neuronal Ceroid Lipofuscinosis,” EMBO Journal 15, no. 19 (1996): 5240–5245. 8895569 PMC452268

[56] L. Ahtiainen , J. Kolikova , A. L. Mutka , et al., “Palmitoyl Protein Thioesterase 1 (Ppt1)‐Deficient Mouse Neurons Show Alterations in Cholesterol Metabolism and Calcium Homeostasis Prior to Synaptic Dysfunction,” Neurobiology of Disease 28, no. 1 (2007): 52–64. 17656100 10.1016/j.nbd.2007.06.012

[57] J. Weng , S. Liu , Q. Zhou , et al., “Intratumoral PPT1‐Positive Macrophages Determine Immunosuppressive Contexture and Immunotherapy Response in Hepatocellular Carcinoma,” Journal for Immunotherapy of Cancer 11, no. 6 (2023): e006655. 37385725 10.1136/jitc-2022-006655 PMC10314632

[58] J. Zhong , Y. Xie , Z. Zhao , X. Chen , J. Fu , and W. Lin , “PTGDS Deficiency as a Driver of M2 Macrophage Polarization and Immunosuppressive Microenvironment in Esophageal Squamous Cell Carcinoma: An Experimental Study,” International Journal of Surgery 111, no. 8 (2025): 5058–5070. 40497781 10.1097/JS9.0000000000002589

[59] Q. Xiang , F. Tian , J. Xu , X. Du , S. Zhang , and L. Liu , “New Insight Into Dyslipidemia‐Induced Cellular Senescence in Atherosclerosis,” Biological Reviews of the Cambridge Philosophical Society 97, no. 5 (2022): 1844–1867. 35569818 10.1111/brv.12866 PMC9541442

[60] Z. Su , Z. Zong , J. Deng , et al., “Lipid Metabolism in Cartilage Development, Degeneration, and Regeneration,” Nutrients 14, no. 19 (2022): 3984. 36235637 10.3390/nu14193984 PMC9570753

[61] M. Ouimet , V. Franklin , E. Mak , X. Liao , I. Tabas , and Y. L. Marcel , “Autophagy Regulates Cholesterol Efflux From Macrophage Foam Cells via Lysosomal Acid Lipase,” Cell Metabolism 13, no. 6 (2011): 655–667. 21641547 10.1016/j.cmet.2011.03.023 PMC3257518

[62] H. Tai , Z. Wang , H. Gong , et al., “Autophagy Impairment With Lysosomal and Mitochondrial Dysfunction Is an Important Characteristic of Oxidative Stress‐Induced Senescence,” Autophagy 13, no. 1 (2017): 99–113. 27791464 10.1080/15548627.2016.1247143 PMC5240829

[63] S. Hamsanathan and A. U. Gurkar , “Lipids as Regulators of Cellular Senescence,” Frontiers in Physiology 13 (2022): 796850. 35370799 10.3389/fphys.2022.796850 PMC8965560

[64] X. Zhang , Y. Qin , X. Wan , et al., “Rosuvastatin Exerts Anti‐Atherosclerotic Effects by Improving Macrophage‐Related Foam Cell Formation and Polarization Conversion via Mediating Autophagic Activities,” Journal of Translational Medicine 19, no. 1 (2021): 62. 33568202 10.1186/s12967-021-02727-3 PMC7877030

