# Supplementary material for: Macrophage Lipid Homeostasis Drives IVDD via a Senescence‐Dependent Impairment of Efferocytosis
Source: Cell Prolif. 2026 Aug 19:e70271. Online ahead of print. doi: 10.1111/cpr.70271 (PMC13490779; doi:10.1111/cpr.70271)
Supplement: Supplementary file 1 [file CPR-9999-e70271-s001.docx]

Supplementary Fig 1A: Venn diagram illustrating the common genes between DEGs and LMGs; B: LASSO coefficient curve plot; C: LASSO partial likelihood deviation plot; D: ROC curve of the LASSO model; E: Risk scores of the control group and IVDD group; F-H: Summary plots of Catboost, LightGBM, and XGboost; I-K: Swarm plots of Catboost, LightGBM, and XGboost.

Supplementary Fig 2A: KEGG pathway enrichment plot of the TIAM2 gene; B: KEGG pathway enrichment plot of the SLC44A4 gene; C: KEGG pathway enrichment plot of the PTGDS gene; D: KEGG pathway enrichment plot of the PPT1 gene.

Supplementary Fig 3A: Bubble plot of marker genes of different intervertebral disc cell subgroups; B: Heatmap of marker gene expression in each macrophage subgroup.

Supplementary Fig 4A: Proportion distribution of various macrophage subgroups; B: Expression profiles of marker genes of macrophage subgroups; C: Volcano plot of differentially expressed genes grouped by lipid metabolism level; D: KEGG enrichment pathway analysis of differentially expressed genes grouped by lipid metabolism level.

Supplementary Fig 5A: Box plot of PPT1 and TIAM2 expression in macrophages with different phenotypes; B: Bubble plot of key lipid metabolism genes expressed in different macrophage subgroups.

Supplementary Fig 6A: Schematic diagram of flow sorting of rat intervertebral disc tissues; B: Polarization fluorescence images of sorted macrophages from rat intervertebral discs of each group; C: Quantitative analysis of macrophage polarization fluorescence images.

Supplementary Table 1 The detailed information of Imaging data

| Donor | Age | Gender | Pfirrmann grade | Diagnosis |
| --- | --- | --- | --- | --- |
| 1 | 43 | Female | Ⅰ-Ⅲ | TCS |
| 2 | 47 | Male | Ⅰ-Ⅱ | TCS |
| 3 | 62 | Male | Ⅱ-Ⅳ | LSS |
| 4 | 76 | Male | Ⅱ-Ⅴ | LDH |
| 5 | 53 | Female | Ⅰ-Ⅳ | LDH |
| 6 | 35 | Female | Ⅰ-Ⅴ | LDH |
| 7 | 64 | Male | Ⅰ-Ⅴ | LSS |
| 8 | 67 | Male | Ⅱ-Ⅳ | LDH |
| 9 | 81 | Male | Ⅱ-Ⅴ | LSS |
| 10 | 41 | Female | 1-Ⅳ | LSS |
| 11 | 65 | Male | Ⅰ-Ⅳ | LDH |
| 12 | 34 | Male | Ⅰ-Ⅴ | LDH |
| 13 | 76 | Female | Ⅱ-Ⅳ | LSS |
| 14 | 79 | Female | Ⅰ-Ⅴ | LDH |
| 15 | 43 | Male | Ⅱ-Ⅴ | LSS |
| 16 | 75 | Female | Ⅰ-Ⅴ | LDH |
| 17 | 62 | Female | Ⅱ-Ⅴ | LSS |
| 18 | 35 | Male | Ⅰ-Ⅴ | LSS |
| 19 | 56 | Male | Ⅰ-Ⅴ | LSS |
| 20 | 34 | Female | Ⅰ-Ⅳ | LDH |
| 21 | 69 | Male | Ⅱ-Ⅴ | LSS |
| 22 | 68 | Female | Ⅱ-Ⅳ | LSS |
| 23 | 53 | Male | Ⅰ-Ⅴ | LDH |
| 24 | 57 | Female | Ⅰ-Ⅳ | LSS |
| 25 | 76 | Male | Ⅱ-Ⅴ | LSS |
| 26 | 82 | Male | Ⅲ-Ⅴ | LDH |
| 27 | 62 | Female | Ⅰ-Ⅳ | LSS |
| 28 | 53 | Female | Ⅱ-Ⅴ | LSS |
| 29 | 66 | Male | Ⅰ-Ⅳ | LDH |
| 30 | 67 | Male | Ⅱ-Ⅳ | LSS |

LDH, lumbar disc herniation; LSS, lumbar spinal stenosis;TCS,tethered cord syndrome

Supplementary Table 2 The detailed information of Blood routine test results

| Donor | **Age** | **Gender** | **Diagnosis** | **Surgical segment** | **Pfirrmann grade** | **Group** |
| --- | --- | --- | --- | --- | --- | --- |
| 1 | 52 | Male | LDH | L4/5 | IV | Severe |
| 2 | 75 | Female | LDH | L4/5 | III | Mild |
| 3 | 77 | Male | LSS | L5/S1 | III | Mild |
| 4 | 38 | Male | LDH | L4/5 | V | Severe |
| 5 | 78 | Female | LDH | L3/4 | III | Mild |
| 6 | 74 | Female | LDH | L4/5 | III | Mild |
| 7 | 64 | Male | LSS | L4/5 | V | Severe |
| 8 | 70 | Male | LDH | L4/5 | V | Severe |
| 9 | 89 | Male | LDH | L5/S1 | III | Mild |
| 10 | 63 | Female | LDH | L4/5 | IV | Severe |
| 11 | 31 | Male | LSS | L4/5 | V | Severe |
| 12 | 88 | Female | LDH | L4/5 | III | Mild |
| 13 | 45 | Female | LSS | L5/S1 | V | Severe |
| 14 | 53 | Male | LSS | L3/4 | IV | Severe |
| 15 | 77 | Male | LSS | L3/4 | V | Severe |
| 16 | 58 | Male | LSS | L4/5 | V | Severe |
| 17 | 83 | Male | LDH | L5/S1 | III | Mild |
| 18 | 62 | Male | LSS | L3/4 | IV | Severe |
| 19 | 33 | Male | LSS | L4/5 | V | Severe |
| 20 | 60 | Male | LSS | L3/4 | IV | Severe |
| 21 | 52 | Female | LDH | L4/5 | V | Severe |
| 22 | 64 | Female | LDH | L3/4 | III | Mild |
| 23 | 65 | Female | LDH | L4/5 | V | Severe |
| 24 | 50 | Female | LDH | L4/5 | V | Severe |
| 25 | 42 | Female | LDH | L3/4 | V | Severe |
| 26 | 60 | Male | LDH | L4/5 | IV | Severe |
| 27 | 85 | Female | LSS | L5/S1 | III | Mild |
| 28 | 45 | Female | LDH | L5/S1 | V | Severe |
| 29 | 55 | Male | LSS | L4/5 | IV | Severe |
| 30 | 58 | Female | LSS | L4/5 | IV | Severe |
| 31 | 42 | Male | LSS | L4/5 | V | Severe |
| 32 | 47 | Male | LDH | L3/4 | V | Severe |
| 33 | 68 | Female | LDH | L4/5 | III | Mild |
| 34 | 62 | Female | LSS | L5/S1 | IV | Severe |
| 35 | 54 | Female | LSS | L4/5 | IV | Severe |
| 36 | 53 | Female | LSS | L5/S1 | IV | Severe |
| 37 | 62 | Male | LDH | L4/5 | IV | Severe |
| 38 | 49 | Female | LSS | L4/5 | V | Severe |
| 39 | 74 | Female | LDH | L5/S1 | III | Mild |
| 40 | 61 | Male | LSS | L5/S1 | IV | Severe |
| 41 | 54 | Female | LDH | L4/5 | IV | Severe |
| 42 | 52 | Male | LSS | L4/5 | IV | Severe |
| 43 | 82 | Male | LSS | L4/5 | III | Mild |
| 44 | 82 | Male | LDH | L4/5 | III | Mild |
| 45 | 39 | Male | LSS | L4/5 | V | Severe |
| 46 | 39 | Male | LSS | L3/4 | V | Severe |
| 47 | 58 | Male | LDH | L4/5 | IV | Severe |
| 48 | 79 | Female | LDH | L4/5 | III | Mild |
| 49 | 63 | Female | LSS | L3/4 | IV | Severe |
| 50 | 48 | Male | LSS | L4/5 | V | Severe |
| 51 | 84 | Female | LSS | L4/5 | V | Severe |
| 52 | 53 | Female | LSS | L4/5 | IV | Severe |
| 53 | 65 | Female | LSS | L4/5 | III | Mild |
| 54 | 56 | Female | LDH | L4/5 | V | Severe |
| 55 | 79 | Female | LDH | L5/S1 | III | Mild |
| 56 | 41 | Male | LDH | L4/5 | V | Severe |
| 57 | 83 | Male | LSS | L4/5 | III | Mild |
| 58 | 74 | Male | LSS | L4/5 | III | Mild |
| 59 | 43 | Female | LDH | L5/S1 | V | Severe |
| 60 | 85 | Male | LDH | L4/5 | III | Mild |
| 61 | 64 | Male | LSS | L5/S1 | III | Mild |
| 62 | 81 | Female | LDH | L4/5 | III | Mild |
| 63 | 78 | Female | LDH | L4/5 | III | Mild |
| 64 | 87 | Female | LDH | L4/5 | III | Mild |
| 65 | 88 | Female | LSS | L4/5 | V | Severe |
| 66 | 49 | Male | LSS | L3/4 | V | Severe |
| 67 | 48 | Female | LSS | L4/5 | V | Severe |
| 68 | 65 | Female | LSS | L3/4 | III | Mild |
| 69 | 77 | Male | LSS | L4/5 | III | Mild |
| 70 | 31 | Female | LSS | L4/5 | V | Severe |
| 71 | 52 | Male | LSS | L4/5 | IV | Severe |
| 72 | 67 | Male | LSS | L3/4 | III | Mild |
| 73 | 61 | Male | LSS | L4/5 | IV | Severe |
| 74 | 36 | Female | LSS | L4/5 | V | Severe |
| 75 | 76 | Male | LSS | L4/5 | V | Severe |
| 76 | 56 | Female | LSS | L5/S1 | IV | Severe |
| 77 | 38 | Male | LSS | L5/S1 | V | Severe |
| 78 | 89 | Female | LDH | L4/5 | III | Mild |
| 79 | 63 | Female | LSS | L5/S1 | IV | Severe |
| 80 | 64 | Male | LSS | L4/5 | III | Mild |
| 81 | 79 | Female | LSS | L4/5 | V | Severe |
| 82 | 31 | Female | LSS | L5/S1 | III | Mild |
| 83 | 39 | Female | LSS | L5/S1 | V | Severe |
| 84 | 34 | Male | LSS | L5/S1 | V | Severe |
| 85 | 47 | Male | LDH | L4/5 | V | Severe |
| 86 | 42 | Male | LSS | L4/5 | V | Severe |
| 87 | 58 | Female | LDH | L5/S1 | V | Severe |
| 88 | 60 | Female | LDH | L4/5 | IV | Severe |
| 89 | 54 | Female | LDH | L5/S1 | IV | Severe |
| 90 | 60 | Female | LSS | L5/S1 | IV | Severe |
| 91 | 44 | Male | LSS | L4/5 | V | Severe |
| 92 | 56 | Female | LDH | L5/S1 | IV | Severe |
| 93 | 74 | Male | LSS | L5/S1 | III | Mild |
| 94 | 85 | Male | LDH | L5/S1 | III | Mild |
| 95 | 53 | Female | LDH | L4/5 | IV | Severe |
| 96 | 66 | Male | LDH | L4/5 | III | Mild |

LDH, lumbar disc herniation; LSS, lumbar spinal stenosis;TCS,tethered cord syndrome

Supplementary Table 3 The detailed information of intervertebral disc samples

| Donor | **Age** | **Gender** | **Diagnosis** | **Surgical segment** | **Pfirrmann grade** | **Group** |
| --- | --- | --- | --- | --- | --- | --- |
| 1 | 58 | Female | LDH | L5/S1 | V | Severe |
| 2 | 31 | Female | LSS | L5/S1 | III | Mild |
| 3 | 77 | Male | LSS | L3/4 | V | Severe |
| 4 | 58 | Male | LSS | L4/5 | V | Severe |
| 5 | 85 | Male | LDH | L4/5 | III | Mild |
| 6 | 64 | Male | LSS | L5/S1 | III | Mild |
| 7 | 64 | Male | LSS | L4/5 | III | Mild |
| 8 | 79 | Female | LSS | L4/5 | V | Severe |
| 9 | 31 | Female | LSS | L5/S1 | III | Mild |
| 10 | 39 | Female | LSS | L5/S1 | V | Severe |

LDH, lumbar disc herniation; LSS, lumbar spinal stenosis;TCS,tethered cord syndrome
